# Diet and subsistence in Bronze Age pastoral communities from the southern Russian steppes and the North Caucasus

**DOI:** 10.1371/journal.pone.0239861

**Published:** 2020-10-14

**Authors:** Corina Knipper, Sabine Reinhold, Julia Gresky, Nataliya Berezina, Claudia Gerling, Sandra L. Pichler, Alexandra P. Buzhilova, Anatoly R. Kantorovich, Vladimir E. Maslov, Vladimira G. Petrenko, Sergey V. Lyakhov, Alexey A. Kalmykov, Andrey B. Belinskiy, Svend Hansen, Kurt W. Alt

**Affiliations:** 1 Curt Engelhorn Center Archaeometry, Mannheim, Germany; 2 Eurasia Department, German Archaeological Institute, Berlin, Germany; 3 Department of Natural Sciences, German Archaeological Institute, Berlin, Germany; 4 Research Institute and Museum of Anthropology of Lomonosov Moscow State University, Moscow, Russian Federation; 5 Integrative Prehistory and Archaeological Science IPAS, Basel University, Basel, Switzerland; 6 Department of Archaeology, Faculty of History, Lomonosov Moscow State University, Moscow, Russian Federation; 7 Institute of Archaeology, Russian Academy of Sciences, Moscow, Russian Federation; 8 Heritage Organization Ltd, ‘Nasledie’, Stavropol, Russian Federation; 9 Center of Natural and Cultural Human History, Danube Private University (DPU), Krems-Stein, Austria; University at Buffalo - The State University of New York, UNITED STATES

## Abstract

The flanks of the Caucasus Mountains and the steppe landscape to their north offered highly productive grasslands for Bronze Age herders and their flocks of sheep, goat, and cattle. While the archaeological evidence points to a largely pastoral lifestyle, knowledge regarding the general composition of human diets and their variation across landscapes and during the different phases of the Bronze Age is still restricted. Human and animal skeletal remains from the burial mounds that dominate the archaeological landscape and their stable isotope compositions are major sources of dietary information. Here, we present stable carbon and nitrogen isotope data of bone collagen of 105 human and 50 animal individuals from the 5^th^ millennium BC to the Sarmatian period, with a strong focus on the Bronze Age and its cultural units including Maykop, Yamnaya, Novotitorovskaya, North Caucasian, Catacomb, post-Catacomb and late Bronze Age groups. The samples comprise all inhumations with sufficient bone preservation from five burial mound sites and a flat grave cemetery as well as subsamples from three further sites. They represent the Caucasus Mountains in the south, the piedmont zone and Kuban steppe with humid steppe and forest vegetation to its north, and more arid regions in the Caspian steppe. The stable isotope compositions of the bone collagen of humans and animals varied across the study area and reflect regional diversity in environmental conditions and diets. The data agree with meat, milk, and/or dairy products from domesticated herbivores, especially from sheep and goats having contributed substantially to human diets, as it is common for a largely pastoral economy. This observation is also in correspondence with the faunal remains observed in the graves and offerings of animals in the mound shells. In addition, foodstuffs with elevated carbon and nitrogen isotope values, such as meat of unweaned animals, fish, or plants, also contributed to human diets, especially among communities living in the more arid landscapes. The regional distinction of the animal and human data with few outliers points to mobility radii that were largely concentrated within the environmental zones in which the respective sites are located. In general, dietary variation among the cultural entities as well as regarding age, sex and archaeologically indicated social status is only weakly reflected. There is, however, some indication for a dietary shift during the Early Bronze Age Maykop period.

## Introduction

Grassland-adapted animal husbandry was one of the most efficient economic strategies of prehistoric communities in Eurasia. Pastoralists relied on the vast resources of steppe environments and profited from close interdependencies with their animals [1]. They were counterpoints to sedentary farming groups with agro-pastoral subsistence practices, i.e. economies based on crop cultivation and localized, small-scale herding. Not only the daily requirements of animal forage, but also the ecological diversity and varying productivity of grasslands encouraged highly flexible and mobile lifestyles with large operating ranges [2–4], which also stimulated cultural interactions [5].

The Bronze Age in the Caucasus represents an outstanding context for studying prehistoric pastoral communities. In particular, the northern flanks of this high mountain system formed an important link between the Near East and the Eurasian steppes and played a crucial role in the dispersal of technologies, innovations, and people [6, 7]. Thousands of kurgans and numerous flat grave cemeteries dominate the archaeological record of the area. Human and animal skeletal remains from burial contexts represent essential sources of spatially and chronologically nuanced information regarding social practices and economic strategies, especially since settlement sites are largely unknown between the late 4^th^ and the mid-2^nd^ millennium BC [8]. Collecting and synthesizing evidence for human and animal dietary compositions is a central aspect of modern archaeological research, and stable carbon and nitrogen isotope analysis (δ^13^C and δ^15^N) of bone collagen contributes increasingly to such investigations [9, 10]. Some existing studies already focused on the north Caucasus and attested to complex entanglements between environmentally influenced isotopic compositions of the plants at the base of the food webs as well as distinct dietary habits and economic strategies [11–17].

The present study focusses on exploring variation among burial communities. Our earliest human samples date to the Eneolithic (n = 2; c. 4330 to 4050 cal. BCE), 90 individuals represent different phases of the Bronze Age (c. 3900/3800 to c. 1000 cal. BCE), ten samples are from the Early Iron Age (c. 1000 to 500 cal. BCE) or later periods, while three could not be dated based on their archaeological contexts. Fifty animal bones served as comparative samples (Bronze Age: n = 43; likely Bronze Age, but not securely dated: n = 5; Iron Age: n = 2).

**Fig 1:**
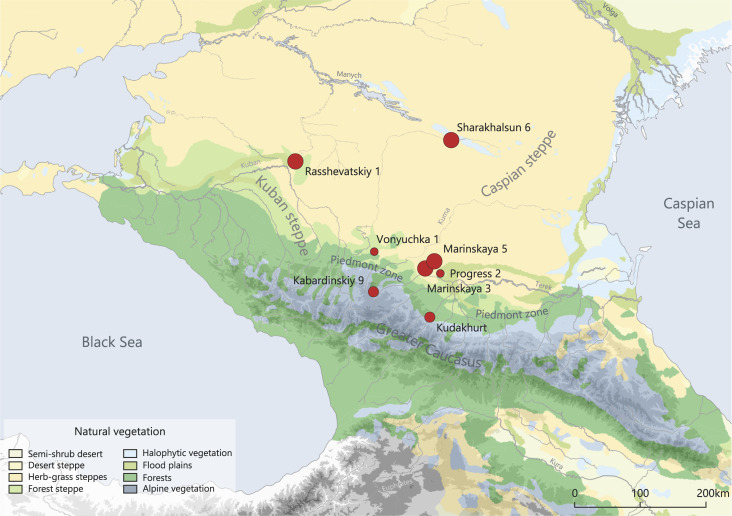
Map showing major landscapes, vegetation zones and investigated sites in the North Caucasus region. The symbol sizes for the sites reflect the number of samples investigated. Background map after {Stone, 2003 #9972}. This dataset is openly shared, without restriction, in accordance with the NASA Data and Information Policy.

Key sites comprise six burial mounds and one flat grave cemetery, all of which contained human inhumations and animal bones from several phases and different archaeological cultures of the Bronze Age. The study aimed at investigating human diets and their variation over time, among different regions as well as variability regarding age, sex, and archaeologically indicated social status. The mounds are located in the piedmont zone and steppe plains north of the Caucasus Mountains, whereas one of the mound sites and the flat grave cemetery represent the upland plateaus (Fig 1).

Based on human bone collagen and animal comparative samples, our study pursues the following objectives:

1. explore environmentally dependent differences of the carbon and nitrogen isotopic composition along food chains;
2. use this information to deduce variation in human dietary habits among regions and over time;
3. explore dietary distinction within burial communities and their relation to age, sex, and potential social differentiation.

## The archaeological context

### Environmental conditions and types of subsistence strategies

This investigation deals with the North Caucasus region in southern Russia between the Black Sea and the Caspian Sea and focusses particularly on the Stavropol region (Fig 1). The study area is environmentally diverse and comprises several biomes, i.e. communities of plants and animals living under the same conditions, and ecotones, the transition zones between them [18]. The most elevated sites are situated in the Caucasus Mountains. The limestone plateaus are hotspots of biodiversity that comprise high quality grassland, ideally suited as pastures [19]. The piedmont zone north of the mountains is a rolling, mosaic-like landscape. It embraces large grazing potential, but also well-watered and fertile soils that were suited for farming. Towards the north, the piedmont zone extents gradually into the vast plains of the southern Russian steppes [20]. Directly adjacent to the piedmont zone is the Kuban herb and forest steppe. Humidity levels decrease towards the north and the east, so that the Caspian steppe with its grasses gradually develops into a semi-desert environment.

Despite their ecological diversity, all these landscapes offered a broad spectrum of pastures with excellent conditions for a pastoral economy [8, 11, 20]. The archaeological record of the 5^th^ to the 2^nd^ millennium BCE suggests that the development and spread of this economic system was a non-linear process and that agro-pastoralism, pastoralism and the exploitation of wild resources oscillated over time [21, 22]. Agro-pastoral communities combined the cultivation of staple crops such as cereals and pulses with domestic animal husbandry of sheep, goats, and cattle as well as exploiting smaller shares of wild resources. This subsistence practice developed in the Levant during the 8^th^ to 7^th^ millennium BCE [23]. Subsequently, it spread across Eurasia and became the dominant economic practice in temperate regions. Agro-pastoral communities were usually sedentary, and stock keeping either concentrated in the hinterlands of the settlements or involved seasonal mobility and transhumance. Farmers consumed a mixed diet with varying proportions of plant food and animal products, such as meat, milk, and dairy products, among which the vegetable component generally dominated [24–26].

In contrast, pastoral communities were more mobile and dominant in the Eurasian steppes. Meat, fat, and dairy products from domestic animals encompassed most of the produced food items, despite the general availability of cultivated crops [27–31]. A wide range of edible wild plants from steppe biomes contributed carbohydrates and vitamins [32–35]. Similar to agricultural communities, the dietary composition of pastoralist societies may have varied among groups in different environments and on an individual basis [15, 36]. In the north Caucasus, the varied biomes allowed for an oscillation between agricultural and pastoral economic strategies, which probably contributed to the absence of permanent habitation sites during major parts of the Bronze Age.

### Archaeological cultures and their economies

In the wider north Caucasus area, first indication for a Neolithic economy, including domestic animals, has been proposed for the Lower Don River in the 7^th^ and 6^th^ millennium BCE, but the evidence is much debated [29, 37, 38]. Archaeological remains of the Darkveti-Meshoko Eneolithic of the mid-5^th^ millennium BCE point to agro-pastoral communities at the northern flanks of the Caucasus (S1 Fig). Settlement features revealed evidence for cultivated crops as well as domestic cattle, sheep, goats, and surprisingly many pigs [39, 40], and aDNA data attested to a paleogenetic component associated with populations in the Fertile Crescent [7].

The first burial mounds between the Caucasus, the Caspian Sea, and the lower Don and Volga Rivers date to the Eneolithic of the late 5^th^ millennium BCE [41]. Some of these graves contained bones of domesticated animals, whereas most bone artifacts represent wild species [20, 18]. Despite the evidence for domesticated animals, it is still unclear whether the communities who erected the earliest mounds in the southern Russian steppe represent the first pastoralists or late hunter-gatherers [42, 43].

During the 4^th^ millennium BCE, settled groups who represented the Early Bronze Age Maykop culture expanded rapidly in the Caucasus piedmonts and the piedmont steppe (3700–2900 cal. BCE). Cultivated crops and domesticated animals from burial and settlement contexts point to a sedentary lifestyle and agro-pastoral subsistence strategies [28, 44] with a strong focus on animal husbandry, especially of cattle [22, 22–23]. The Maykop period transformed the north Caucasus into an anthropogenic landscape with monumental burial mounds that attest to outstanding individuals and an increasing social differentiation. Moreover, late Maykop sites yielded the first evidence of draught animals and wheeled vehicles [45]. In addition to the sites in the piedmont area, there are numerous burials with Maykop elements, the so-called “Steppe Maykop” further north, in the grass steppe and semi-desert environments. These sites have been interpreted as evidence of seasonal pastoral mobility from the piedmonts into the Caspian steppe [20, 238–241]. Recent ancient DNA studies, however, suggest remarkable genetic differences between the Maykop groups in the piedmont zone and the “Steppe Maykop” populations [7].

During the Middle Bronze Age, people started to use the mountain plateaus of the North Caucasus, while the archaeological record in the lowlands became regionally differentiated. Settlements were abandoned and burial mounds become the only archaeological sources [46]. In the steppe zone, people of the Yamnaya culture both continued to use some of the Steppe Maykop burial mounds and established new ones around 3300 cal. BCE. From 2800 cal. BCE on, Catacomb burials succeeded those of the Yamnaya culture [20]. In the Kuban steppe and in the piedmont steppe of the Stavropol region, the Middle Bronze Age includes the Yamnaya, Novotitorovskaya, Catacomb and Northcaucasian archaeological cultures. Many burial mounds contained graves associated with several of these, often with considerable chronological overlap. Bones of domestic animals, traces of mobile architecture with the simultaneous lack of evidence for permanent settlements, and remains of wheeled vehicles attest to certain aspects of a mobile lifestyle, which are well aligned with pastoralism being the dominating subsistence strategy [20, 47].

The increasing aridity of the 4.2 kyr climate event brought an end to the Catacomb groups in the steppe and the piedmonts. Thereafter, post-Catacomb and late Bronze Age communities, such as Lola groups and their piedmont counterparts developed under adverse climatic conditions. In the 17^th^ century BCE, the steppe appears almost devoid of people. At the same time, people began to resettle the mountain plateaus above the forest belt [46, 48]. During the second half of the 2^nd^ millennium BCE, these groups developed a complex mountain agricultural economy based on a mixed economy with intensive seasonal herding. Burials were placed into already existing mounds or into flat grave cemeteries, most of which were located in the uplands.

### Climatic conditions

Today, the climate in the North Caucasus is temperate in the plains and piedmonts and alpine in the mountains. It also becomes increasingly continental from West to East. Humidity decreases to the north, from the piedmont area across the Kuban steppe into the drier Caspian steppe and the semi-desert near the Manych River. The interplay of the Westerlies, the Monsoon and the Siberian anticyclone governs the climatic regimes [49, 50]. During the Holocene, these components shifted repeatedly, which resulted in shifts of vegetation zones as well as the ecologic and economic potential of the different steppe environments.

Despite the lack of high-resolution paleoclimate data from the study area itself [20, 219–222, 44, 71–77, 51], data from neighboring regions point to comparatively dry conditions during the late 5^th^ and the 4^th^ millennium BCE [49, 52–54]. This trend correlates with the first so-called ‘Holocene rapid climate event’ between 6.4 and 5 kyr that triggered comparatively arid and cool conditions throughout the northern hemisphere [55]. During the 5^th^ millennium BCE, broad leave forests expanded in the West Caucasus Mountains, whereas on-site pollen-records from Eneolithic and Early Bronze Age Maykop settlements indicate steppe-like grasslands in the piedmont zone [39, 56]. After 3100 cal. BCE, people of the Yamnaya, North Caucasian, and Catacomb archaeological cultures experienced more humid and stable conditions [20, 220]. Severe climatic stress affected the populations at the end of the Middle Bronze Age. Between 2200 and 2000 cal. BCE, the 4.2 kyr event, a short period of extremely dry and cold climate recorded in many parts of the Northern hemisphere, dominated vast areas of Western Asia and the Mediterranean. It probably resulted from a shift of the inter-tropical convergence zone and an increasing impact of the westerlies [55]. The climatic incursion of aridity, cooling, hyper-salination of soils and considerable erosion is also recorded in soils underneath mounds of the late Catacomb and post-Catacomb cultures, in the north Caucasus steppes [57]. These fluctuations in climate caused environmental stress and overgrazing, which negatively affected the stability of the populations in the Near East, the Mediterranean, and in the Caucasus [50, 99–114]. The sharp decline of the number and size of archaeological sites indicates a severe crisis of the steppe economy including significant depopulation after 2200 BCE [20, 221–222]. Small communities characterize the post-Catacomb and Late Bronze Age 1 (LBA 1) horizons, whereas by the 17^th^ century BCE, the entire steppe and piedmont areas at the foot of the Caucasus Mountains were apparently abandoned [46, p. 14–16].

## Principles of stable carbon and nitrogen stable isotope analysis and previous studies in the North Caucasus

Stable isotope analysis of bone collagen is a well-established method for palaeodietary reconstruction [58, 59]. Nitrogen in bone collagen derives from the protein fraction of the human diet, whereas carbohydrates and fats do not contain this element. Carbon in collagen originates to about 75% from dietary protein, while carbohydrates and fats contribute the remaining quarter [60, 61]. Isotope fractionation causes differences in the stable isotope compositions of carbon (δ^13^C) and nitrogen (δ^15^N) of the major food groups and along food chains [62]. Major distinctions of the δ^13^C values occur between plants of the C_3_ and C_4_ photosynthetic pathways as well as between terrestrial and marine dietary components. The δ^13^C values of modern C_3_ plants range from about -37 ‰ to -20 ‰ with an average of -27 ‰ [63, 64]. C_4_ plants exhibit δ^13^C values between -16 and -9 ‰, averaging -12.5 ‰ [65, 66]. Fossil fuel combustion and deforestation caused the δ^13^C values of atmospheric CO_2_ to drop from -6.5 ‰ to -8 ‰ between 1850 and today, and this shift of -1.5 ‰ has to be considered when interpreting carbon isotope compositions of archaeological samples [67, 68]. Delta^13^C values of bone collagen of primary consumers are about 5 ‰ higher than the δ^13^C values of the plants they eat and increase by 0 to 2 ‰ with every further trophic level [69]. Typical δ^13^C values of bone collagen of preindustrial herbivores average at about -21.5 ‰ for pure C_3_ plant consumers and -6 ‰ for pure C_4_ plant consumers. Delta^13^C values of above -18 ‰ are considered a benchmark of C_4_ plant contribution [70]. Environmental properties, such as humidity levels [63, 64] and forest cover [71] cause minor variations within the δ^13^C spectrum of C_3_ plants. Marine foodstuffs also have elevated δ^13^C values similar to the range of C_4_ plants. Freshwater fish usually exhibit lower δ^13^C values between about -25 and -18 ‰ with considerable interregional variation [72–75] which may also include elevated δ^13^C values of about -15 to -12 ‰ as found in the Siberian forest steppe [76].

In the north Caucasus, C_3_ plants predominate in the temperate environments of the mountain plateaus, the piedmont zone and the Kuban steppe. The drier portions of the Caspian steppe may have supported significant proportions of C_4_ plants, probably with varying shares over time and due to climate fluctuations [17].

Nitrogen isotope variation is primarily caused by the trophic level effect. Isotope fractionation along the food chain causes the δ^15^N values to increase by about 3 to 5 ‰ with each trophic level [77] or up to 6 ‰ for humans as clinical studies suggest [78]. Therefore, nitrogen isotope ratios reflect the contribution of meat and dairy products, but also of fish to the human diet [77, 79]. However, data interpretation is not always straightforward, and needs to consider variation of the isotopic composition of the plants at the base of the food webs. Their nitrogen isotope ratios may be influenced by environmental properties, such as aridity [80, 81], or reflect the application of animal manure, which raises the δ^15^N values remarkably [82, 83].

The north Caucasus has been subject to previous carbon and nitrogen stable isotope analyses. Selected individuals of several sites and archaeological cultures revealed first indications of chronological and spatial variation of bone collagen isotopic compositions [11]. Substantial research has been conducted in connection with radiocarbon dating, where light stable isotope ratios were explored regarding reservoir effects and fish consumption [14, 16]. Stable isotope analyses were also included early on in the investigation of the archaeological remains from the Caspian steppe and the drier areas to its north, where they contributed significantly to our current picture of lifestyle and subsistence [15, 20]. Moreover, an extensive study of plant and animal samples aimed at overcoming the limitations of the explanatory power of human collagen data alone [17]. The flat grave cemetery of Klin Yar provided the first extensive C and N isotope dataset from a Caucasus upland plateau and attested to dietary change from the Early Iron Age to the Early Medieval period [13]. In the south Caucasus mountains [84, 85] and in the steppe areas beyond the north Caucasus, stable carbon and nitrogen isotope data helped exploring Bronze Age subsistence strategies, in Kazakhstan [31, 36, 86, 87], the Samara micro-region in the Volga area [88], the southern Ural mountains [89], and in Siberia [76]. Along with the results of recent ancient DNA analyses [7, 90, 91], these studies form a solid framework for the interpretation of the data presented here.

## Investigated sites and sampled material

During most of the Bronze Age in the northern Caucasus and in the steppe, burial mounds (‘kurgans’) with well-preserved human and animal remains dominate the archaeological record, whereas settlement features are scarce. Skeletal remains are therefore a crucial source of information regarding diet and subsistence. This study focusses on the Stavropol region in the southern Russian Federation. From an enormous collection of about 2500 Bronze Age burials that have been excavated over the last two decades, we selected the inhumations of five mounds, a flat grave cemetery, and Eneolithic burials from two multi-component sites for stable C and N isotope analysis. The locations represent the Caucasus Mountains, the piedmont zone, the humid Kuban forest steppe and the dry Caspian Steppe (Fig 1). From the mounds, we included all human individuals with sufficient bone preservation. This comprehensive sampling distinguishes the present study from the selective approach of previous investigations [11, 14, 16].

Overall, we extracted collagen from 105 human and 50 animal bones for comparison (Tables 1 and 2, S1A and S1B Table, S2 Fig). Sampling of human bones concentrated on ribs and used other bones only if no ribs were available. Most of the animal bones belonged to domestic sheep/goat or cattle and were either directly associated with the human burials or were deposited in separate units (so-called “ritual complexes”) in the embankments of the mounds. Some faunal samples originated from contemporary mounds in the same mound groups.

**Table 1:** Investigated sites and corresponding number of sampled human individuals grouped according to archaeological cultures and phases of the Bronze Age (*collagen extraction or quality criteria failed).

	Eneolithic	Early Bronze Age		Middle Bronze Age			Late Bronze Age	Iron Age	Not dated	Not sampled
	Eneolithic	Maykop	Novotitorovskaya	Yamnaya	North Caucasian	Catacomb	Late Bronze Age	Iron Age/ Sarmatian or later		
		3900–2900 cal. BCE	3300–2800 cal. BCE	3300–2400 cal. BCE	2800-2400/2200 cal. BCE	2500–2200 cal. BCE	1700–1000 cal. BCE	1000-600/6th cent. BC-4th cent. AD or later		
**Caspian steppe:** Sharakhalsun 6 Kurgan 2		5 this study; 1 Shishlina 2008		6		5				Graves 10 and 12
**Kuban steppe:** Rasshevatskiy 1 Kurgan 21			6 (+3*)	2	2	2 (+2*)		1		Graves: 1, 3, 4, 6, 9, 12, 14
**Piedmont zone:** Marinskaya 3 Kurgan 1		1			3 (+1*)	9		2	1	Graves 2, 11
**Piedmont zone:** Marinskaya 5 Kurgan 1		6			15	1	1 (+1*)	6	2	Graves 21, 31
**Caucasus Mountains:** Kabardinskiy 9					6		5	1		
**Caucasus Mountains:** Kudakhurt 14							7			most of cemetery sampled by K. Fuchs
**Piedmont zone:** Progress 2	1									
**Piedmont zone:** Vonyuchka	1									

**Table 2:** Investigated sites and corresponding number of sampled human individuals grouped according to age classes and sex of adult individuals, number of animal samples, and number of samples for radiocarbon dating and dendrochronology.

Site and number of human samples	Infans I	Infans II	Juvenile	Adult male	Adult female	Adult indet.	Animal bones	Absolute dating
**Caspian steppe:** Sharakhalsun 6 Kurgan 2 (n = 18)	4	1		10	3		n = 26; sheep/goat, cattle, horse, dog, birds, hare (some from other mounds at the same site)	n = 11 (^14^C)
**Kuban steppe:** Rasshevatskiy 1 Kurgan 21 (n = 18)	3	2	1	5	3	4	n = 10; some from other mounds at the same site sheep, goat, dog (some from other mounds at the same site)	n = 3 (^14^C)
**Piedmont zone:** Marinskaya 3 Kurgan 1 (n = 17)	1		2	11	1	2		n = 3 (^14^C), n = 1 (dendrochronology)
**Piedmont zone:** Marinskaya 5 Kurgan 1 (n = 32)	3	2	4	17	6		n = 5; cattle, sheep/goat	n = 7 (^14^C), n = 3 (dendrochronology)
**Caucasus Mountains:** Kabardinskiy 9 (n = 12)	1	1		7		3	n = 2; sheep/goat	n = 6 (^14^C)
**Caucasus Mountains:** Kudakhurt 14 (n = 7)	1	1		4		1		n = 1 (^14^C)
**Piedmont zone:** Progress 2 (n = 1)				1			n = 7; sheep/goat	
**Piedmont zone:** Vonyuchka (n = 1)					1			

The term “adult” summarizes individuals of above 18 years of age. For more detailed information on ages at death and results of absolute dating see S1A Table, animal samples are listed in S1B Table.

The chronologically earliest samples comprise two individuals of the Eneolithic period (c. 4330 to c. 4050 BC). They are subsamples from the larger sites of **Vonyuchka (= Konstantinovskaya 1;** VON) and **Progress 2** (PROG2) in the piedmont zone [7, 92, 93] (S3 Fig). Progress 2 also provided Bronze Age animal bones.

The sites of **Sharakhalsun** are among the densest clusters of burial mounds in the Kalmykian Caspian steppe. Sharakhalsun 6 is a West-East oriented line of kurgans, which runs parallel to the right bank of the Kalaus River near its outlet into the Manych River. We sampled all burials with sufficient bone preservation from mound 2 (Sharakhalsun 6/Kurgan 2 = SHAR). The mound measured 50 m in diameter, was three meters high and excavated in 2001 [94]. Two Early and one Late Maykop construction layers contained seven inhumations of the 4^th^ millennium BCE. Yamnaya groups added four graves in the centre and one in the periphery of the mound during the 3^rd^ millennium BCE. The youngest burials are represented by five Late Catacomb inhumations. Four graves of the Maykop, Yamnaya, and Catacomb cultures contained wooden wagons [45, 87–89, 95]. In all, the sample from Sharakhalsun comprised 17 human individuals and 26 animal bones. 11 bones and wood samples were radiocarbon dated. Hollund et al. [11, Tab. 2] and Shishlina [20, Tab. 20] report previously generated stable isotope data for a child in Maykop grave 17, which we include in our considerations.

**Rasshevatskiy** (site 1/mound 21; RASS) is located in the Kuban steppe. Rasshevatskiy 1 is a W-E-oriented line of 27 mounds extending over 2 km. Mound 21 was excavated in 2000. It had an oval shape, extended over 110 x 85 m, was up to 6.4 m high and comprised five mound shells of different sedimentary compositions [96, 97]. The oldest grave in the mound dates to the Steppe Maykop culture but was too badly preserved for sampling. The subsequent inhumations have been assigned to the Yamnaya (n = 3), Novotitorovskaya (n = 5), North Caucasian (n = 2), and Catacomb archaeological cultures (n = 8). Burials in the mound terminated in the medieval period (grave 5). Notable are dismantled wagons of the Late Catacomb Culture (grave 8) and Novotitorovskaya Culture (grave 7 containing 5 individuals). The isotope samples comprised 18 human individuals and ten animal bones from this and other mounds at the site. Human bone samples from three of the burials were radiocarbon dated.

**Marinskaya 3** (MAR3) is a cemetery of three large kurgans and several ploughed down mounds in the piedmont zone, about 40 km NE of the initial rises of the Caucasus Mountains. Mound 1 was excavated in 2007. It was about 40 m in diameter, four meters high, and surrounded by a circular, 2.5 m deep ditch [98]. The first mound shell was erected over a rich and undisturbed Maykop grave (grave 18) with a paved floor, plastered and painted walls and a wooden ceiling, which was dated to 3304±25 BC by dendrochronology. A stone construction and an earthen mound covered the grave. During the Middle Bronze Age, four graves of the North Caucasian culture and eight graves of the Catacomb culture were dug into the mound. The final inhumations in MAR3 date to the Early Iron Age (grave 1) and the Sarmatian period (grave 4 = 10). The isotope sample comprised 17 human individuals. Three radiocarbon and one dendrochronological date inform on the absolute chronological placement.

**Marinskaya 5** (MAR5) is a single mound of 34–40 m in diameter and 4.3 m high, situated about 1 km SW of MAR3 and surrounded by a ditch. It was excavated in 2009 [45, 99]. The initial constructions include four mound shells with six inhumations of the Maykop period. They comprise a sequence of three inhumations of the Early and three inhumations of the Late Maykop period dating to the first, respectively the second half of the 4^th^ millennium BC. We use the terms 'Early' and 'Late' Maykop, and are aware that the later Maykop complexes traditionally would be labeled 'Maykop-Novosvobodnaya' [44, 100].

About 600 years later, 17 graves of the North Caucasian culture and one Catacomb grave as well as a fourth mound shell were added. They are succeeded by three graves probably dating to the Late Bronze Age. Two disturbed inhumations likely also date to the Bronze Age. The interments terminated with five Sarmatian graves. Archaeologically significant are bucrania (cattle skulls without mandibles) associated with a richly furnished Late Maykop inhumation (grave 25) [45] and two North Caucasus burials (graves 19 and 23). We sampled bones from 32 human individuals and five animals. Three dendrochronological and seven radiocarbon dates inform on the absolute chronology of the site.

**Kabardinskiy 9** (KAB) is a cemetery that consists of several burial mounds of the Middle Bronze Age North Caucasus culture (MBA 1) and its final stage (MBA 2) on a Caucasus Mountain plateau [48]. The site was previously labelled 'Kabardinka' [101], but belongs to a larger necropolis recorded as 'Kabardinksiy 9'. Poor bone preservation limited the osteological assessments. The isotope sample comprised twelve human and two animal individuals. Six human bones were radiocarbon dated [48].

**Kudakhurt** (KUD) is a flat grave cemetery in the Caucasus foothills, which comprises 130 burials of the final Middle (MBA 2) and Late Bronze Age (LBA I) [48]. The initial analyses reported here concentrated on a multiple burial (grave 186), from which we analysed six individuals, and one single inhumation (grave 212). An animal bone associated with the multiple burial was radiocarbon dated. A full anthropological assessment of the cemetery including stable carbon and nitrogen isotope is currently undertaken by Katharina Fuchs [102].

## Methods

### Ethics statement

The investigated material has been excavated by the heritage organization Ltd. ‘Nasledie’, Stavropol, the Eurasia Department of the German Archaeological Institute, the Institute of Archaeology of the Caucasus, Nalchik, the Lomonosov Moscow State University or the Institute of Archaeology of the Russian Academy of Sciences. The geographical coordinates and license numbers for the excavations for each of the sites are listed in S1A Table. All skeletons are stored at the heritage organization Ltd. ‘Nasledie’, Stavropol and inventoried according to sites, burial mounds and grave numbers at the archaeological depository at Novopavlovsk. Sampling permissions have been granted by A. Belinskiy and A. Lychagin (Ltd. ‘Nasledie’, former GUP ‘Nasledie’). The material was exported with permission issued by the Cultural Ministry of the region of Stavropol.

### Sample preparation, analysis, and data evaluation

Collagen extraction and analysis of most samples were conducted at the University of Mainz and the Curt Engelhorn Center Archaeometry gGmbH Mannheim, Germany. Some animal bones were prepared and analysed at the University of Basel, Switzerland. Sample preparation at all three institutions followed the same protocol [103] with modifications as described in [104]. Compact bone portions were cut and the surfaces removed. Between 200 and 800 mg of sample were demineralized in 10 ml of 0.5 N HCl at initially 4°C and later at room temperature for 14 days, rinsed to neutrality and reacted with 10 ml of 0.1 M NaOH for 24 h at 4°C, rinsed again to neutrality and gelatinized in 4 ml of acidified H2O (pH 2–3) for 48 h at 75°C. Insoluble particles were separated using EZEE filter separators, and the collagen frozen and lyophilized. Analysis was conducted in duplicates. About 1.5 mg of collagen were placed into tin boats and loaded into the autosampler of an elemental analyser (vario EL III, Elementar Analysensysteme) coupled to an IsoPrime High Performance Stable Isotope Ratio Mass Spectrometer (GV Instruments) at the Institute for Organic Chemistry at the University of Mainz or with a Thermo Flash 2000 Organic Elemental Analyzer coupled to a Thermo Finnigan Mat 253 Mass spectrometer at the Department for Applied and Analytical Palaeontology at the University of Mainz. Analysis at the Department of Environmental Sciences, University of Basel was performed using an INTEGRA2 EA-IRMS instrument (Sercon Ltd., Crewe, UK).

Isotope compositions were reported in δ-notation in per mil relative to VPDB for carbon and AIR for nitrogen. Data produced at the University of Mainz were corrected using two-point calibrations based on USGS 40 and IAEA N2 or USGS 41 for nitrogen and USGS 40 and USGS 41 or CH6 and CH7 and for carbon [105]. Reproducibility of internal and external standards was better than ± 0.2 ‰ for nitrogen and ± 0.1 ‰ for carbon.

At Basel, raw nitrogen and carbon isotope data were blank-, linearity, and drift-corrected and then normalized to the AIR and VPDB (Vienna Pee Dee Belemnite) scales, respectively, by means of two-point calibrations based on EDTA (in-house standard) and IAEA N2 or EDTA and IAEA CH6, respectively. Nitrogen and carbon isotopic compositions are reported in δ-notation as δ^15^N and δ^13^C in per mil relative to AIR and VPDB (Vienna Pee Dee Belemnite), respectively. Reproducibility of internal and external standards was better than ± 0.25 ‰ for δ^15^N and better than ± 0.1 ‰ for δ^13^C.

Data evaluation and statistical analysis have been performed in Microsoft Excel and SigmaPlot (Version 14.0). Because of the fossil fuel effect that lowers the δ^13^C values of modern plants, we added 1.5 ‰ to the published data [17] that we used for considerations about preindustrial food webs [68]. Ancient plant data do not require this correction. For estimations of δ^13^C values at the first trophic level, we considered an isotopic enrichment of 5 ‰ between the carbon isotope ratios of the plants and the bone collagen values of herbivores [67, 69]. Further up the food chain, we considered an average increase of the collagen δ^13^C values by 0.8 ‰ and the collagen δ^15^N values by 4 ‰ between adjacent trophic levels [80]. The extent of trophic enrichment may, however, vary among species and depend on the protein content or type of food [69, 77, 106, 107]. This causes uncertainty in predicting potential collagen isotope compositions of herbivores based on plant baseline data and at the higher trophic levels.

## Results

### Collagen quality

Ninety-seven human samples processed in this study yielded collagen that fulfilled the quality criteria for ancient collagen as suggested by Ambrose [108] and van Klinken [109]. They yielded between 0.6 and 20.7% of collagen, 22.3 to 44.0% of C, 7.3 to 16.5% of N, and had atomic C/N ratios of between 3.1 and 3.6 (S1A Table). Three samples from RASS did not contain any collagen, and the extracts of two samples from RASS, one from MAR3 and one from MAR5 failed the quality criteria.

Forty-four out of 50 animal samples fulfilled the collagen quality criteria (S1B Table). They yielded between 0.2 and 22.1% of collagen, 35.0 to 44.6% of C, 11.6 to 17.1% of N, and had atomic C/N ratios of between 3.1 and 3.6. Three samples from SHAR, one from RASS, one from MAR5, and one from Progress 2 failed the quality criteria. One sample from SHAR and one from RASS had low collagen yields, whereas all other quality criteria were within acceptable ranges.

### Stable carbon and nitrogen isotope data

#### The human samples

**Fig 2:**
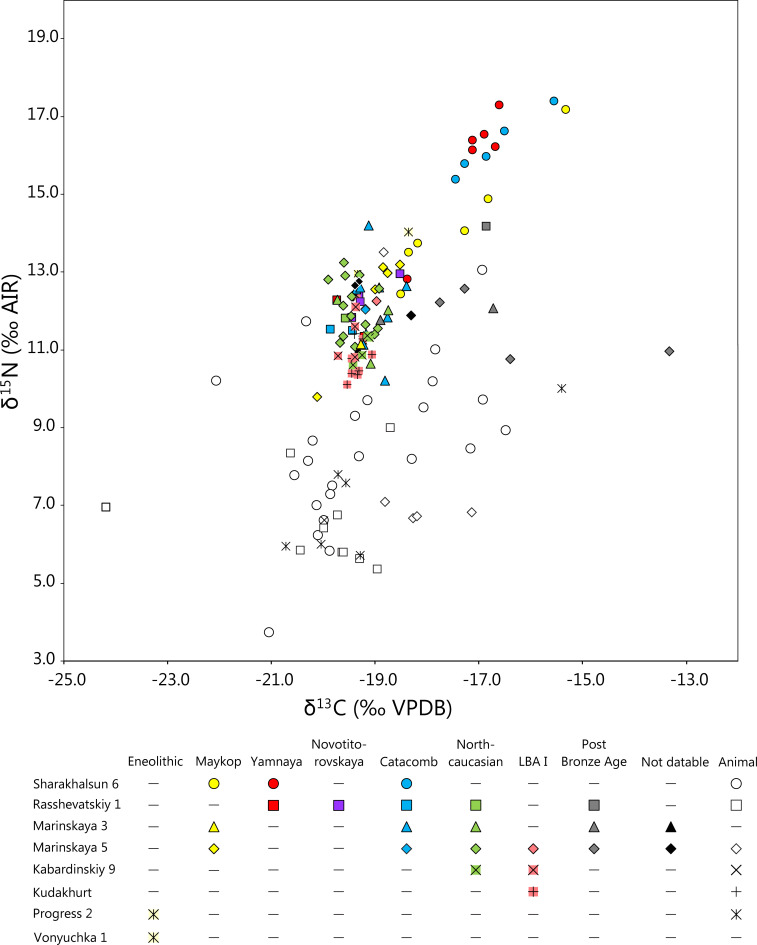
Scatter plot of δ^13^C and δ^15^N values of human and animal collagen investigated in this study. The colours of the symbols denote the archaeological cultures and the shapes mark the sites from which the samples originated.

**Fig 3:**
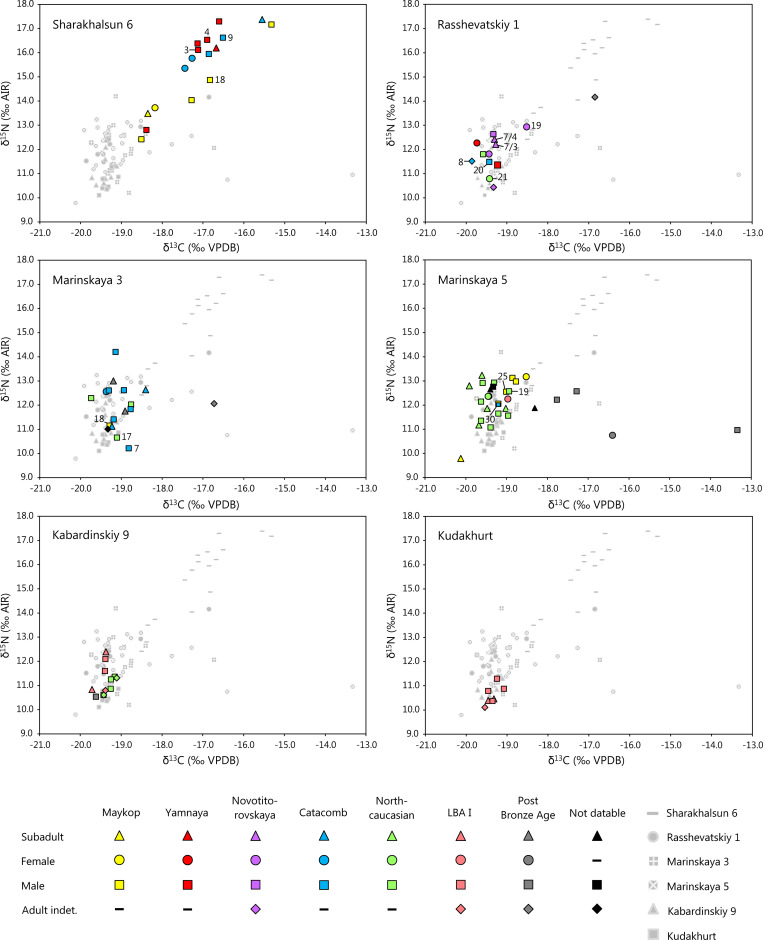
Scatter plots of δ^13^C and δ^15^N values of human and animal collagen investigated in this study grouped according to sites. The colours of the symbols illustrate the archaeological cultures and the shapes mark the age and sex of the sampled individuals. The samples from all other sites are greyed out for comparison. Burials with exceptional grave constructions or grave goods, such as wagons, are numbered.

The δ^13^C and δ^15^N values of all human individuals varied widely between -20.1 and -13.3 ‰ and 9.8 and 17.4 ‰ respectively (Table 3, Figs 2 and 3, S1A Table). A rib of the female MAR5, grave 6 yielded an unusually low δ^15^N value of 7.4 ‰ and a δ^13^C value of -18.6 ‰. The sample was excluded from further evaluation, because the data suggest that it was a mistakenly sampled animal bone.

**Table 3:** Summary statistics of the δ^13^C and δ^15^N values of the human bone collagen investigated in this study.

Site	Landscape	N	δ^13^C Min	δ^13^C Max	δ^13^C Avg.	δ^13^C 1 SD	δ^15^N Min	δ^15^N Max	δ^15^N Avg.	δ^15^N 1 SD
**Sharakhalsun 6**	Caspian steppe	17	-18.5	-15.3	-17.1	0.9	12.4	17.4	15.4	1.6
**Rasshevatskiy 1**	Kuban steppe	12	-19.7	-18.5	-19.4	0.3	10.3	12.9	11.8	0.7
**Marinskaya 3**	Piedmont zone	13	-19.7	-18.4	-19.1	0.3	10.2	14.2	11.9	1.1
**Marinskaya 5**	Piedmont zone	23	-20.1	-18.5	-19.3	0.4	9.8	13.2	12.1	0.8
**Kabardinskiy 9**	Caucasus Mountains	12	-19.7	-19.1	-19.4	0.2	10.5	12.4	11.2	0.6
**Kudakhurt 14**	Caucasus Mountains	7	-19.5	-19.1	-19.3	0.2	10.1	11.3	10.6	0.4
**Progress 2**	Piedmont zone	1			-19.3				13.0	
**Vonyuchka 1**	Piedmont zone	1			-18.4				14.0	

Abbreviations: Min = Minimum, Max = Maximum, Avg. = Average, SD = Standard deviation. δ^13^C values are given in ‰ V-PDB, δ^15^N values are given in ‰ AIR.

The kurgans of MAR3, MAR5 and RASS contained post-Bronze Age burials (i.e. Early Iron Age Koban l, Late Iron Age Sarmatian, Middle Ages), who often had higher δ^13^C values than the Bronze Age samples from the same mounds. At MAR3 and MAR5, the sample size of Bronze Age and post-Bronze Age individuals was large enough for statistical comparison. Their δ^13^C values were significantly different (Mann-Whitney Rank Sum Test (U = 7.000, p < 0.001)), whereas the δ^15^N values were not (two-tailed t-test: t(40) = 0.894, p = 0.377). Because the post-Bronze Age samples may obscure regional trends of isotopic distinction, the following evaluation uses only data of the Eneolithic and the Bronze Age.

*Regional differentiation during the Bronze Age*. Table 3 summarizes the descriptive statistics for the Eneolithic and Bronze Age individuals from each site. Considering all human data, the δ^13^C and δ^15^N values are highly correlated (r^2^ = 0.765). This trend is especially marked for the samples with δ^13^C values of above -19.0 ‰, and much weaker for those with δ^13^C values below -19 ‰.

The burials from Sharakhalsun in the dry Caspian steppe have generally higher δ^13^C and δ^15^N values than those from the more humid locations. According to a Kruskal-Wallis H-test the differences among all sites were significant for both δ^13^C with H(5) = 45.460, p <0.001 and for δ^15^N with H(5) = 50.559, p <0.001. A repeated analysis without the site of Sharakhalsun did not attest to significantly different δ^13^C values (one-way ANOVA (F(4,62) = 1.909, p = 0.120), whereas the δ^15^N values were significantly different (one-way ANOVA (F4,62) = 6.622, p < 0.001). The samples from KUD had significantly lower δ^15^N values than those from MAR5, MAR3 and RASS and those from KAB had lower values than those from MAR5. In general, the individuals from the sites in the Kuban steppe and in the piedmont area had higher δ^15^N values than those from the sites in on the Caucasus upland plateaus.

Overall, the inter-site comparison revealed that the human stable isotope ratios related strongly to the landscapes in which the sites were located. This suggests an influence of environmental conditions on the C and N isotope data. For the following evaluations, we therefore summarized the sites according to landscapes. SHAR represents the dry steppe with a trend to high δ^13^C and δ^15^N values, RASS, MAR3 and MAR5 are summarized as Kuban forest steppe/piedmont area with generally low δ^13^C and intermediate δ^15^N ratios, and KAB and KUD characterize the Caucasus mountains with low values of both isotope ratios.

*Chronological trends during the Bronze Age*. At SHAR in the dry steppe, there was a statistically significant difference of the δ^13^C and δ^15^N values among the Maykop, Yamnaya, and Catacomb burials (two-way ANOVA F(2, 28) = 3.609, p = 0.040). The dissimilarity was due to the Maykop individuals, who had lower average values in both isotope systems than the other two chronological groups. Considering only the Maykop individuals, those of the Early Steppe Maykop period (graves 15, 16, 17) had lower δ^13^C and δ^15^N values than those of the Late Steppe Maykop period (graves 6, 11, 18). The comparison of the δ^13^C and δ^15^N values of the individuals assigned to the Yamnaya and Catacomb cultures did not yield a statistically significant difference (two-way ANOVA F(1, 18) = 0.700, p = 0.414).

The sites of RASS, MAR3, and MAR5 in the humid steppe and piedmont zone included individuals of the Maykop, Novotitorovskaya, Catacomb, North Caucasian and Late Bronze Age I cultural groups. The δ^13^C and δ^15^N values of the representatives of these cultural units were not significantly different (two-way ANOVA F(4, 86) = 0.423, p = 0.791). Fig 3 illustrates the widely overlapping isotope ratios at each of these sites. However, a trend of changing isotope ratios within the Maykop period that was noted for SHAR also appeared at the sites in the Kuban steppe and piedmont area. Burials of the Early Maykop period (graves 32, 33, 34 at MAR 5) formed a narrow cluster of δ^13^C values between -18.9 and -18.5 ‰ and δ^15^N values between 13.0 and 13.2 ‰, whereas the isotope values of the individuals of the Late Maykop period were lower and more variable.

The datable burials at KAB and KUD on the Caucasus Mountain plateaus belong either to the North Caucasian or to the Late Bronze Age I cultures. Neither the δ^13^C nor the δ^15^N values yielded any statistically significant difference between the two chronological groups (two-tailed t-test δ^13^C: t(17) = 1.803, p = 0.0892; δ^15^N: t(17) = 0.116, p = 0.909).

**Fig 4:**
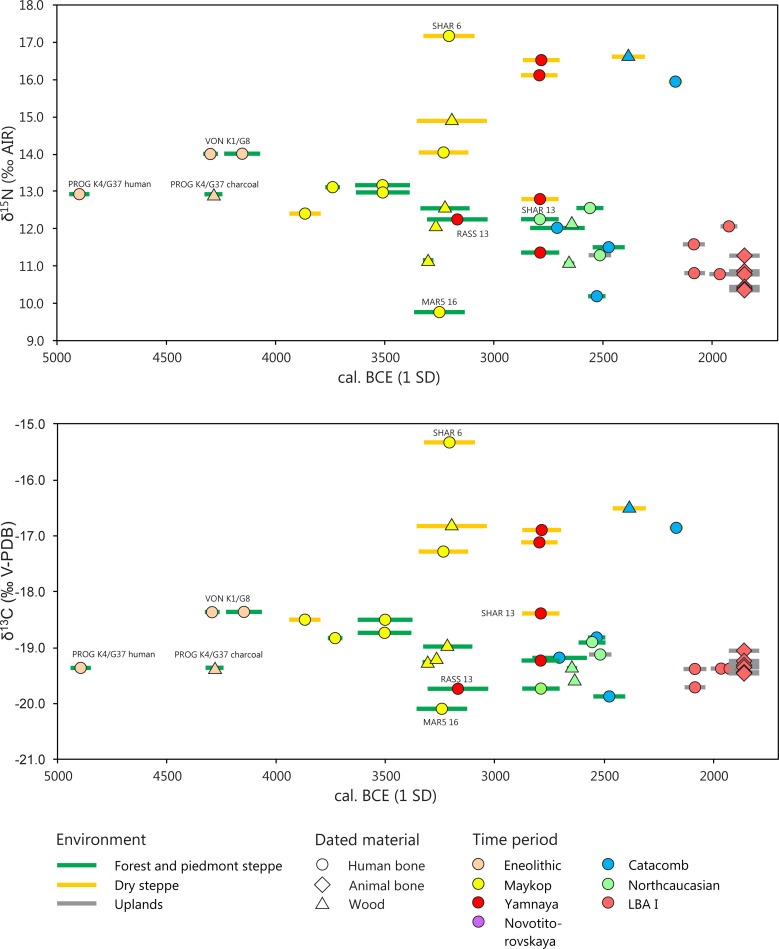
**Stable nitrogen (above) and carbon (below) isotope ratios of human bone collagen associated with radiocarbon dates from the same burial contexts.** The colour of the error bars (1 SD of radiocarbon dates) indicates the landscapes in which the sites are located. Symbol forms indicate the material that was radiocarbon dated and symbol colours mark the archaeological culture of the burials.

Numerous burials have also been radiocarbon dated. The samples come either from the human skeletons themselves, from animal bones, wood of the grave constructions, or grave goods, such as wagons from the same contexts (Fig 4). Earlier analyses of pairs of human bones and non-human samples brought attention to the reservoir effect that leads to apparently older dates for human remains [14, 16, 110]. In the current investigation, an Eneolithic human bone from Progress 2, kurgan 4, grave 37, yielded a radiocarbon date that was several hundred years older (4942–4849 cal. BCE, 1 SD) than the date of a piece of charcoal (4323–4242 cal. BCE, 1 SD) from the same inhumation and confirms the necessity of considering possible reservoir effects.

Fig 4 illustrates that the Eneolithic and Early Maykop burials prior to ca. 3500 cal. BCE had very similar δ^13^C and δ^15^N values. Neither was there remarkable variance over time, nor did the Early Maykop burials exhibit clear regional differences between the piedmont area/Kuban steppe and the Caspian steppe. Among the Late Maykop burials, dating after ca. 3500 cal. BCE, however a tendency emerged of a regional distinction between higher δ^13^C and δ^15^N values in the dry steppe and lower δ^13^C and δ^15^N values in the more humid zones of the steppe and the piedmont area. Among the samples with paired radiocarbon dates and light stable isotope ratios from this study, this trend continued among the Yamnaya, North Caucasian, and Catacomb burials until around 2200 cal. BCE. The final MBA 2 and LBA I between 2000 and 1800 cal. BCE was only represented in the uplands and revealed comparatively low δ^13^C and δ^15^N values that continued the trend seen among the samples from the Kuban steppe and piedmont area.

*Variation according to age and sex*. Sample sizes of individuals of the age groups infans I and infans II/adolescent as well as adult males and females were too low to evaluate age and sex-specific differences at each site over time (Fig 3). We therefore grouped the data according to landscapes as introduced above. At SHAR in the dry steppe, the δ^13^C and δ^15^N values of the subadult individuals fell among those of the adults and did not form a separate group exhibiting the breastfeeding effect [111, 112]. Among the adults, the δ^13^C and δ^15^N values of the females (n = 3) plot among those of the males (n = 10), and do not reveal any indication of sex-specific dietary differences.

At the sites in the Kuban steppe and Piedmont area, the stable isotope values of infans I individuals (n = 5) were also indistinct from those of the older age classes and lacked significant differences to the adult females (Mann-Whitney-Test δ^13^C: U = 16.000, p = 0.622; two-tailed t-test δ^15^N: t(11) = 0.626, p = 0.544). There was also no statistically significant difference between adult males (n = 27) and females (n = 8), even though possible trends may have been obscured by the different sample sizes (two-tailed t-test δ^13^C: t(33) = 0.836, p = 0.409; δ^15^N: t(33) = -0.489, p = 0.628).

Among the small data sets from KAB and KUD on the mountain plateaus, adult females were lacking, and the isotope data of the few subadult individuals fell within the data of the adult males and adult individuals of indeterminate sex.

*Variation according to grave construction and grave goods*. Fig 3 highlights individuals in the isotope scatter plots who had elaborate grave constructions and/or outstanding grave goods indicating social differentiation within the burial communities. At SHAR, four graves of the Maykop (grave 18; S4 Fig), Yamnaya (graves 2 and 4) and Catacomb (grave 9) cultures contained dismantled wagons [45, Fig 8.10–11]. The male of grave 18 belonged to the group of the so called “sitting dead” and revealed multiple healed fractures that had occurred several years prior to his death [95]. The δ^13^C and δ^15^N values of the bone collagen of these four individuals were indistinct from those buried without wagons.

At RASS, five individuals of a multiple burial of the Novotitorovskaya culture (grave 7; S5 Fig) were buried underneath a wagon. Grave 20 (Catacomb culture) contained a wheel, grave 8 (Catacomb culture) yielded amongst other grave goods a golden earring and was accompanied by two wagons, and grave 21 (North Caucasian culture) a silver earring. The stable isotope data of these individuals plot within the main cluster of the site. In contrast, the juvenile female of grave 19, with the highest δ^13^C and δ^15^N values among the Bronze Age individuals, was buried without apparent grave goods (Fig 3).

**Fig 5:**
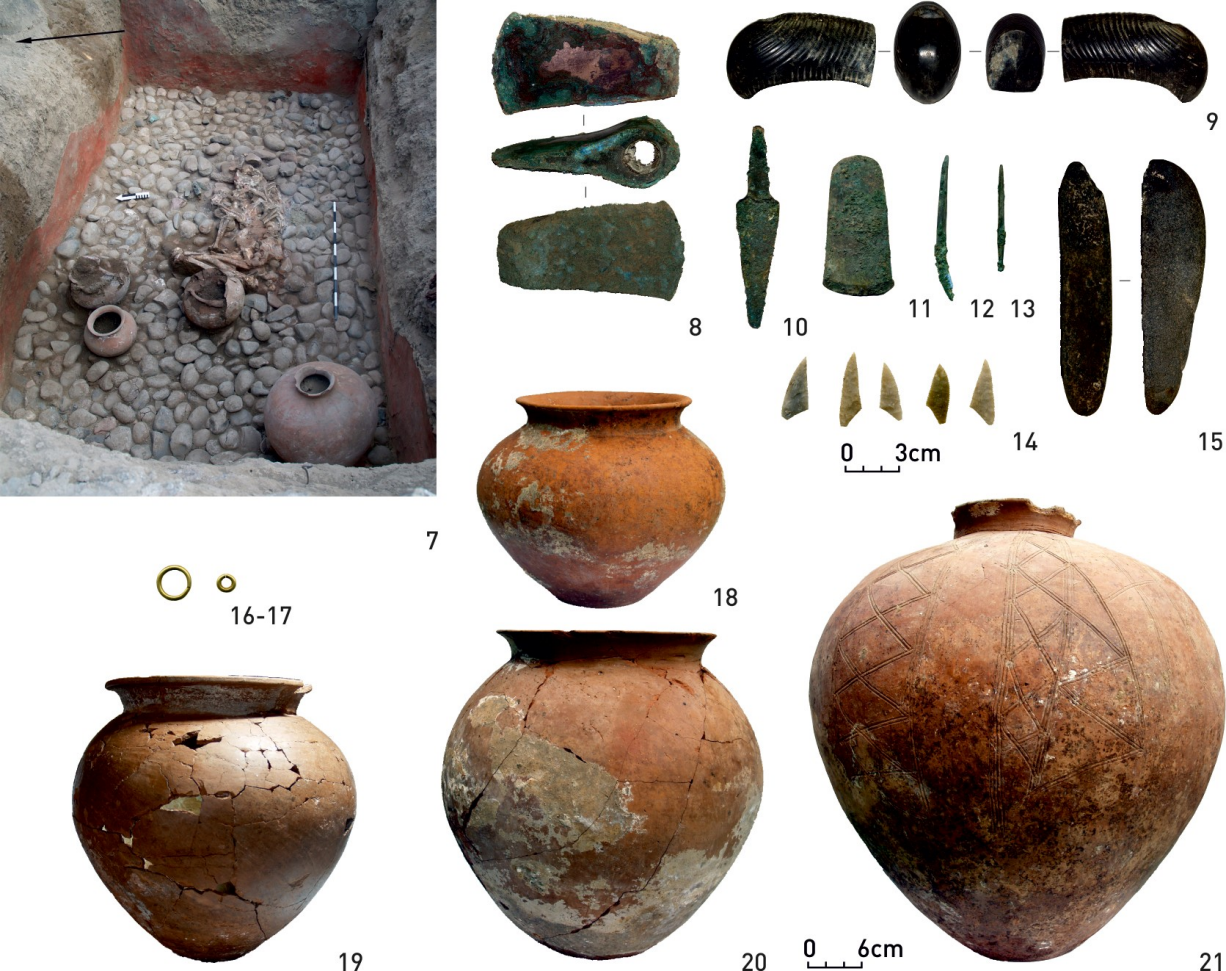
Marinskaya 3, Grave 18. Richly furnished inhumation of the Maykop culture with two golden earrings, a stone sceptre-axe, several bronze objects, four ceramic vessels and seven stone arrowheads.

The primary burial at MAR3 (grave 18) (Fig 5) was a richly furnished inhumation of the Maykop culture furnished with personal ornaments, weapons and ceramic vessels [113]. The Catacomb grave 17 in the same mound was remarkable due to a bronze earring and bronze and faïence beads. Grave 7 contained two bronze earrings, bronze and white faïence beads, and several pendants. The isotope ratios of these individuals plot within the data spectrum of the site and are very similar to each other with comparatively low δ^15^N values.

The kurgan of MAR5 revealed a similar pattern. Remarkable are burials with bucrania (graves 19, 25, 30) of the Maykop (S6 Fig), Catacomb and North Caucasus cultures [45, 81–83, Fig 8.4, 8.6. 8.8]. The bucrania associated grave 25 had looped bronze rings in their noses. Wood remains in front of the skulls may represent a poorly preserved yoke [45]. Grave 25 also contained a golden earring, two bronze daggers, a ceramic vessel, and a bone arrowhead. The isotope data of the collagen of these burials were very similar to each other and within the main cluster of all individuals in this mound.

#### The animal samples

**Fig 6:**
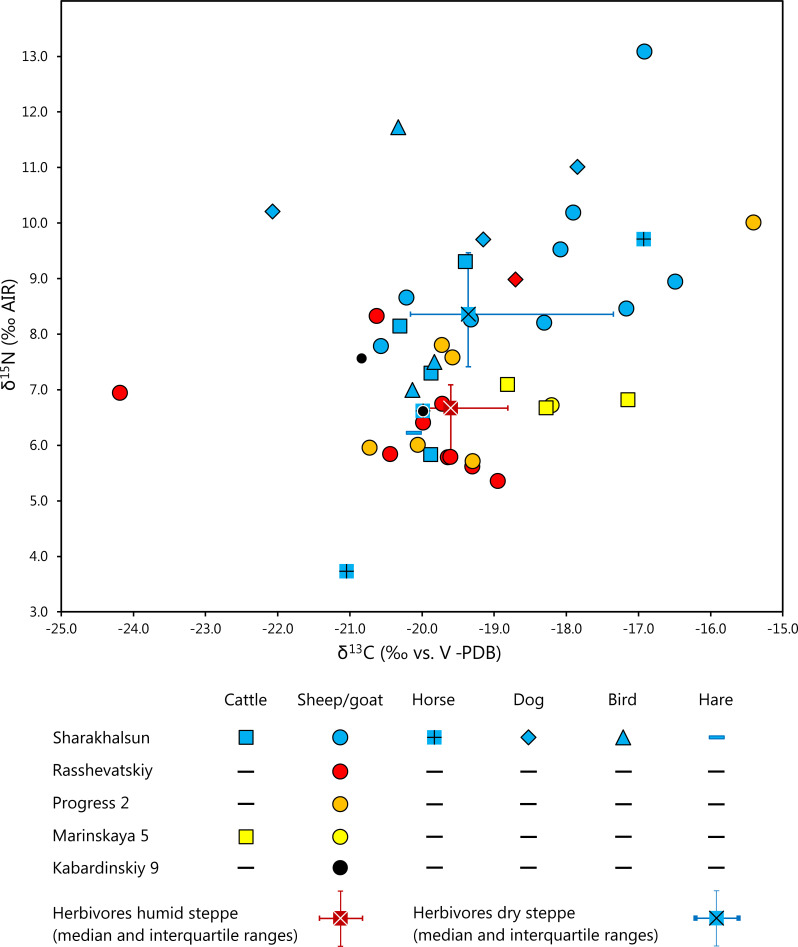
Scatter plot of δ^13^C and δ^15^N values of animal bone collagen investigated in this study. Symbol shapes and colors indicate the animal species and sites from which the samples originated.

Similar to the humans, the collagen extracted from animal bones yielded highly variable stable C and N isotope compositions (Fig 6; S1B Table, S2 Table). Because sample numbers per species and per site were lower than for the humans, we resigned from statistical significance tests based on specific sites. Overall, the δ^13^C values ranged from -24.2 ‰ (RASS, sheep/goat) to -15.4 ‰ (PROG2, sheep/goat), while the δ^15^N values varied between 3.7 ‰ (SHAR, horse) and 13.0 ‰ (SHAR, sheep/goat). The sheep/goat bones from SHAR (dry steppe) had variable and often comparatively high δ^13^C values of between -20.6 and -16.5 (average: -18.3 ± 1.4 ‰; n = 9) and δ^15^N values of between 7.8 and 13.0 ‰ (average: 9.2 ± 1.6 ‰; n = 9). In contrast, the δ^13^C values of the SHAR cattle collagen were more homogenous (range: -20.3 to -19.4 ‰; avg. -19.9 ± 0.4 ‰; n = 4) and covered only the lower end of the data range of the cattle. The δ^15^N values were likewise lower on average (range: 5.8 to 9.3 ‰; avg.: 7.6 ± 1.5 ‰; n = 4). Summarizing all data of the domestic herbivores, including three horse bones, yielded -18.9 ± 1.5 ‰ for δ^13^C and 8.4 ± 2.0 ‰ for δ^15^N (n = 16).

The sheep/goat bones from RASS in the Kuban steppe, exhibited overall lower and more homogenous δ^13^C and δ^15^N values than those from the dry steppe. Excluding one outlier with an unusually low δ^13^C value of -24.2 ‰, the carbon isotope values ranged between -20.6 and -19.0 ‰ (avg.: -20.3 ± 1.6 ‰; all samples included), while the δ^15^N values varied from 5.4 to 18.3 ‰ (avg. 6.3 ± 0.9 ‰). The δ^13^C values of three cattle bones from MAR5 (piedmont zone) ranged from -18.8 to -17.2 ‰ (avg. -18.1 ± 0.9 ‰) while a sheep/goat bone yielded a δ^13^C value of -18.1 ‰. The δ^15^N values of the same samples fell between 6.7 and 7.1 ‰ (avg. 6.9 ± 0.2 ‰) for the cattle and was 6.7 ‰ for the sheep/goat bone. The summarized averages for both herbivore species were -18.1 ± 0.7 ‰ for δ^13^C and 6.8 ± 0.2 ‰ for δ^15^N. The dataset for Progress comprised six sheep/goat bones with δ^13^C values between -20.7 and -15.4 ‰ (avg. -19.1 ± 1.9 ‰) and δ^15^N values between 5.7 and 10.0 ‰ (avg. 7.7 ± 1.7 ‰). One specimen (BZNK 640/1) yielded the highest values in both isotope ratios of both categories. Removal of this outlier changes the averages to -19.9 ± 0.5 ‰ for δ^13^C and 6.6 ± 1.0 ‰ for δ^15^N. Two sheep/goat bones from KAB in the uplands averaged -20.4 ± 0.5 ‰ in δ^13^C and 7.1 ± 0.7 ‰.

## Discussion

### Stable isotope data as indicators of environmental factors and dietary compositions

The slightly rolling to flat steppe landscape north of the Caucasus mountain chain includes vast areas without natural boundaries. However, both the climatic conditions and vegetation change gradually from south to north, from the Caucasus Mountain plateaus, to forests in the piedmonts, open shrub and grass land in the steppe, to almost desert-like conditions in central Kalmykia. The interpretation of the analytical results of human and animal bones from different parts of this landscape must necessarily disentangle environmental influences from different dietary compositions and also regard a potentially mobile lifestyle that provided access to resources from different habitats.

### Regional variation of carbon and nitrogen isotope values at the base of the food webs

**Fig 7:**
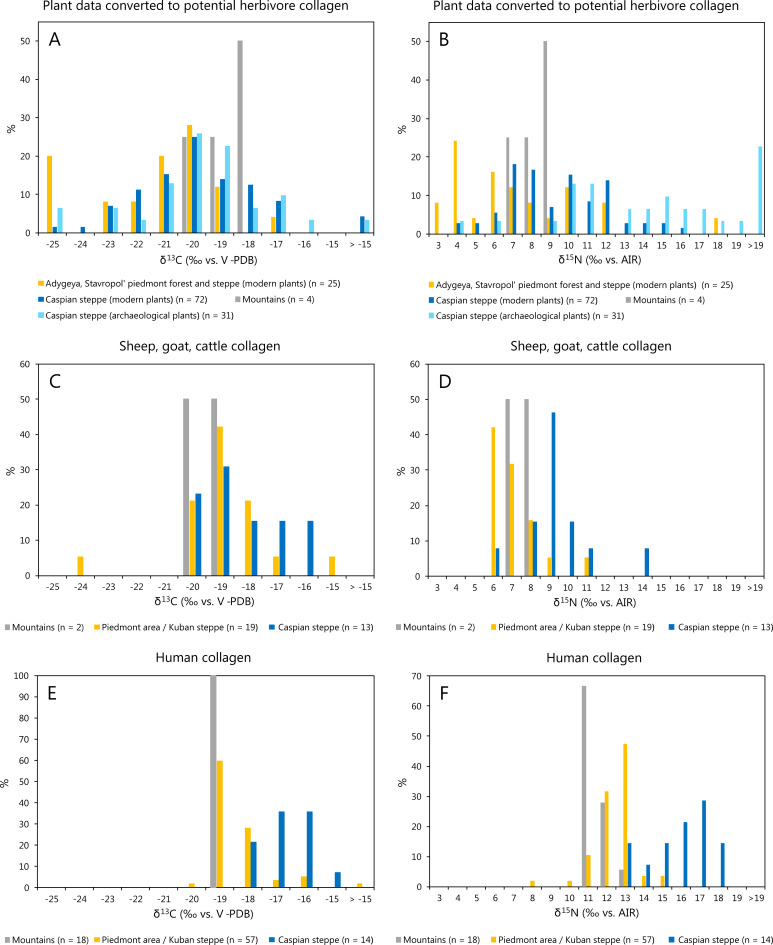
Histograms of δ^13^C and δ^15^N values of modern and archaeological plants converted to potential herbivore collagen (A, B), domestic herbivore collagen (C, D) and human collagen (E, F). Histograms A and B are based on data from Shishlina et al. [17]. Histograms C-F are based on data from this study.

The stable isotope values of the collagen of both animals and humans were highly variable, and the most prominent difference existed between the data from the piedmont area/Kuban steppe and those from the Caspian steppe (Fig 2). First indication for an isotopic distinction between these regions as well as remarkable internal variation was already detected in initial analyses of animal bone collagen from the study area [11]. In order to evaluate to what extent the isotope composition of bone collagen depends on environmental factors or indeed reflects dietary compositions, Shishlina et al. [17] compiled an extensive set of samples of modern and archaeological plants from environmental zones that included parts of our study area and similar landscapes. We used these data to assess whether the herbivores tested in the current investigation likely foraged on plants from those environmental zones in which they were found. The reported localities are grouped to correspond to the three landscapes as outlined above: the Caucasus Mountains (= Stavropol region, mountains), the piedmont zone and Kuban steppe (= Adygeya, piedmont forest, Adygeya steppes, Stavropol Region, steppes and piedmont), and the Caspian steppe (= V. Remontnoye, Caspian Depression, Southern Yergueni Hills, Kuma-Manych Depression Middle Yergueni) [17, Supplementary data, Tab. 3 and 5]. To facilitate the comparison of Shishlina et al.’s [17] data with the animal and human collagen obtained in this study, we added trophic level enrichments of 5 ‰ to the δ^13^C and 4 ‰ to the δ^15^N values of the plants. The data distributions of the resulting potential stable isotope compositions of herbivore collagen were evaluated in histograms (Fig 7A and 7B). The δ^13^C values of archaeological and modern plants (and respective potential animal collagen) from all three landscapes overlap widely and are mainly typical of C_3_ plants in open habitats. Comparatively high δ^13^C values of -24.5 ‰ in the modern plant matter (corresponding to -23.0 ‰ after fossil fuel correction and -18.0 ‰ of herbivore collagen) occurred predominantly in the Caspian steppe and confirmed that δ^13^C values of C_3_ plants tend to increase in hot and dry climatic conditions (cf. [80, 114, 115]). Comparatively low δ^13^C values of around -31.5 ‰ (corresponding to -30.0 ‰ after fossil fuel correction and -25.0 ‰ of herbivore collagen) likely represent the canopy effect on C_3_ plants [71, 116]. They were reported for the Adygeya piedmont forest and occurred only rarely in the Caspian steppe. Few modern and archaeological plants with δ^13^C values of above -16 ‰ (corresponding to -14.5 ‰ after fossil fuel correction and -9.5 ‰ of herbivore collagen) document the occurrence of C_4_ plants in the Caspian steppe [65]. The carbon isotope composition of the few samples obtained from the mountains was typical for C_3_ plant that grew in open habitats.

The δ^15^N values of the modern plants also varied remarkably between -3.8 and 11.5 ‰, which corresponds to 2.8 to 16.5 ‰ in potential herbivore collagen. Despite considerable variation within each regional group and overlaps among the data ranges, the δ^15^N values of the samples from the piedmont forests and humid areas of the steppe were on average lower than those from the Caspian steppe (Fig 7B). The δ^15^N values of the archaeological samples of reed mats and grasses from Bronze Age kurgan burials ranged between -0.5 and 21.5 ‰ (corresponding to 3.5 to 25.5 ‰ in potential herbivore collagen) with numerous of these samples exceeding the isotope ratios of the modern plants [17, Supplementary data Table 5]. The heterogeneity of the isotope compositions of the plants and the remarkably high δ^15^N values among them complicate the interpretation of the isotope data of animal and human collagen significantly. Feeding on plants with high δ^15^N values results in nitrogen isotope compositions of the consumer’s collagen that would otherwise be typical for organisms of higher trophic levels and imply the consumption of meat, dairy products or fish. Moreover, the overlap of the δ^13^C and δ^15^N values of the plants from different landscapes impedes predicting typical values of animals that foraged in the different parts of the study area.

### Carbon and nitrogen isotope values of the animal bones

For the Caucasus mountains, the sample sizes for animal collagen and published plant data are small compared to the other landscapes. The δ^13^C values of the collagen from KAB were indistinguishable from those recorded for herbivores from the piedmont zone/Kuban steppe and the Caspian steppe (Fig 7C). Regarding the δ^15^N values, they fell into the range of those from the piedmont area/Kuban steppe (Fig 7D). Moreover, estimations of herbivore stable isotope ratios as based on plants [17] and measured collagen values were in good agreement.

**Fig 8:**
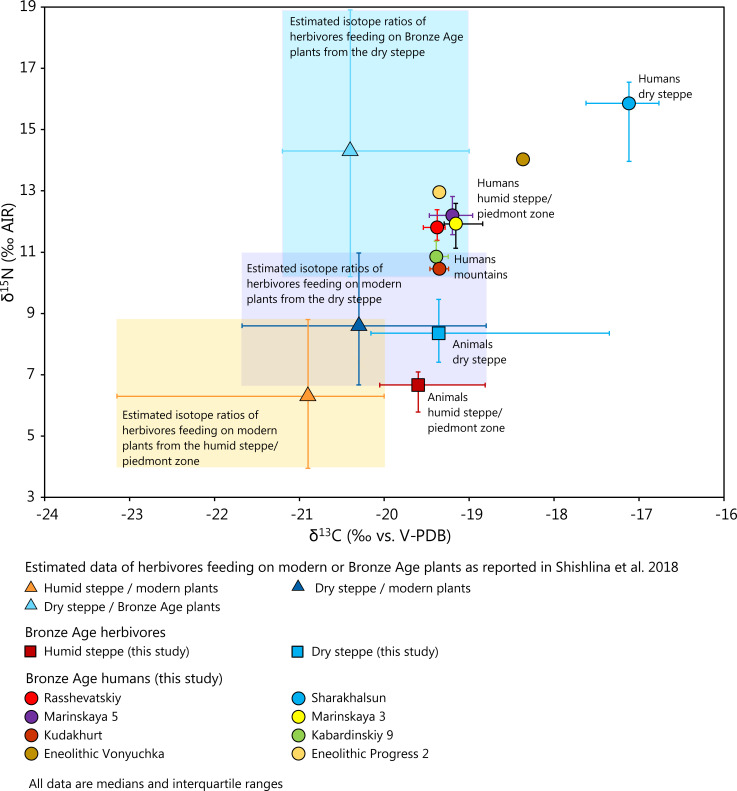
Medians and interquartile ranges of δ^13^C and δ^15^N values of animal bone collagen from the humid and dry steppe and humans grouped according to sites. The shaded areas indicate potential medians and interquartile ranges of herbivores feeding on modern or archaeological plants from different portions of the steppe (Plant data after: Shishlina et al. [17]).

The stable isotope ratios of the animals from the humid areas in the southern parts of the steppe differed slightly according to their find locations. Most of the δ^13^C values of the sheep and goat collagen from RASS and PROG corresponded to the upper half of the carbon isotope ratios predicted from measurements on modern vegetation that grew in the piedmont area or humid steppe [17] (Figs 6, 7A, 7D and 8). This finding suggests that C_3_ plants from open landscapes formed the predominant forage of the tested specimens [71, 77, 117]. One sample of goat collagen from RASS appeared to be an outlier with a low δ^13^C value of -24.2 ‰ (BZNK 251/1; Late Catacomb culture). It indicates an animal that fed on plants whose isotopic composition differed from those of the forage of the other animals at the site and possibly represents a different habitat. Notably, similar δ^13^C values down to below -25 ‰ occurred frequently among the estimated collagen values based on modern plant samples (Fig 7A), especially in the Adygeya piedmont forest, where they likely reflect the canopy effect of the forested habitats [116].

Considering the data distribution among the modern and archaeological plant samples, it is surprising that δ^13^C values of below -20 ‰ did not occur more frequently among the tested animal collagen. This observation suggests that the animal forage was generally limited in the variety of species and exploited habitats as compared to the potential complete spectrum of data found in vegetation in the piedmonts and humid steppe. Changes of the isotopic composition of plants over time due to anthropogenic influences, environmental parameters or climate change may have widened or shifted the range of isotopic composition of plant matter over time. Furthermore, the animal collagen also averages dietary carbon that was taken up over months or years, which reduces the range of variation of the collagen data compared to the isotope data of the plants. The low δ^13^C value of BZNK 251/1 from RASS may suggest that forested or more humid parts of the landscape existed around the site, but were usually avoided for grazing. This animal may also have originated elsewhere and reached RASS through herd mobility, peaceful exchange, or raiding of opposing groups. The sheep BZNK 640/1 from PROG2 is another possible example for such interregional connections. Its δ^13^C value of -15.4 ‰ implies substantial contributions of C_4_ plants, which, according to the data presented by [17], did not occur in the piedmont area, but north of it, i.e., in the drier Caspian steppe. Therefore, the result points to a non-local origin of this animal also.

At MAR, which lies less than 20 km away from Progress, all four herbivore samples had higher δ^13^C values than those observed at RASS and PROG. Their values of between about -19 and -17 ‰ were at the upper limit or above the range of isotope values of herbivores feeding on C_3_ plants from open habitats and rather point to some contribution of C_4_ plant matter. Again, at least the one sample with the highest value (BZNK 275/1) points to some input of C_4_ plants and implies herding ranges beyond the humid steppe and piedmont area. Notably, this sample represents one of two bucrania with nose rings associated with Maykop grave 25 at MAR5 (S6 Fig).

Most of the δ^15^N values of the animals from the sites in the piedmont zone and humid steppe are in the range of those from temperate climates, similar to those from central Europe [118, 119]. They also correspond to δ^15^N values of herbivores as estimated from δ^15^N values of modern plants in ecologically similar landscapes in the North Caucasus (Fig 8) [17], but only cover a part of this data spectrum (Fig 7B and 7D). Similar to the considerations regarding δ^13^C, long-term averaging of the isotopic signals, varying dietary values of the sampled plants, and suitability of the sampled localities for grazing likely contributed to narrowing the range of the δ^15^N values of the animal collagen compared to the δ^15^N values of the plants. The highest δ^15^N value–BZNK 640/1, the same sheep from PROG2 as discussed above–complements the apparently exotic δ^13^C value and underlines the possible non-local origin of the animal.

At SHAR in the dry Caspian steppe, both the δ^13^C and the δ^15^N values of the herbivore collagen were in the range of those from the other landscapes or even higher. The δ^13^C values point to foraging on C_3_ plants from open habitats and some contribution of C_4_ plants. The data range overlapped with the estimates based on modern and archaeological plants [17] (Figs 7A–7D and 8). Similar to the findings from the piedmont zone and the Kuban steppe, δ^13^C values of below around -20 ‰ might have been expected based on the modern vegetation data, but were missing among the collagen values of the archaeological sheep, goat and cattle. The δ^15^N values were also within the range to be expected from the regional plant data [17], but, once again, only represent part of it (Figs 7B, 7D and 8). Especially the archaeological plants from sites in the Caspian steppe yielded numerous exceptionally high δ^15^N values. Having foraged on these plants should have led to even higher δ^15^N values among the animals than observed among our collagen samples. Overall, the δ^15^N data of the animal collagen were in parts similar to δ^15^N values of herbivores from sites in temperate climates [118, 119 9511], whereas numerous samples yielded higher δ^15^N values. The currently published data confirm that these and even remarkably higher values could have been derived from foraging on plants from the Caspian steppe [17].

At SHAR, the stable isotope composition of the collagen samples of the animals also differed among species (Fig 6). The δ^13^C values of the cattle bones were restricted to a range of 0.9 ‰ (-20.3 to -19.4 ‰) and point to preferential feeding on C_3_ plants growing in open habitats. The small range is remarkable, considering the large variation among the plant data [17]. In contrast, the δ^13^C values of the small ruminant bones varied more widely (-20.6 to -16.5 ‰; range: 4.1 ‰) and reflect a dietary spectrum ranging from a predominance of C_3_ plants from open habitats to notable contributions of C_4_ plants. Differentiation among the herbivore species at SHAR is also evident from the nitrogen isotope compositions. The cattle bones yielded lower and more variable values (5.8 to 9.3 ‰; range 3.5 ‰) than those of the sheep/goat (7.8 to 10.2 ‰; range 2.4 ‰; with an outlier with δ^15^N = 13.0 ‰ excluded). The isotope distinction between small and large ruminants indicates that either the different domestic animal species were herded on different pastures or that they fed selectively on different plants available in the same habitats. Examples of elevated δ^13^C values may indicate that pastoralists foddered animals with cultivated millet as it has been recently discussed for Bronze Age contexts in Kazakhstan [120].

Three dog bones from Sharakhalsun averaged higher in their δ^15^N values than the herbivores, which is consistent with an omnivorous diet. The dogs’ δ^13^C values also varied widely. They included the lowest δ^13^C value, which may point to the contribution of some freshwater fish as a possible source of ^13^C-depleted carbon [76, 88]. Three bird bones of unknown species have δ^13^C values typical for feeding on C_3_ plants in open habitats, whereas their variable δ^15^N values suggest different contributions of animal-derived or plant foodstuffs with different N isotope ratios [17].

In summary, especially the isotope data of the small ruminants tend to differentiate between the more humid and dryer sections of the study area. This observation suggests that mobility ranges of the animal herds did not regularly include both of these major environmental zones. At SHAR, cattle herding strategies may have differed from those for sheep and goats, with some of the small ruminants consuming larger shares of forage from the dry steppe. Individual isotopic outliers, as identified at PROG and RASS, indicate an exchange of animals, which may it have been peaceful or not, and interregional contacts among groups who herded their animals in different landscapes. The animal bone collagen from MAR5 seems unusual for a piedmont steppe site, as the elevated δ^13^C values indicate some contribution of C_4_ plants, which are uncommon in this area. Future research on a larger sample size of different species will evaluated whether foddering cultivated C_4_ plants to domestic animals or mobility away from the piedmont zone are more likely explanations.

Overall, the animal data can serve as a general characterisation of the carbon and nitrogen isotope composition of collagen of herbivores at sites in different landscapes and provide a baseline for the animal-derived foodstuffs of the human diet. However, differences among species, site-specific deviations from the average values of a landscape, and individual outliers also confirm that domestic animals depended on human agency. Besides serving as baseline values, the data may reflect cultural overprint as well as herding strategies and interregional exchange. The different focal areas of the distribution of the available data of archaeological and modern plants document that the environmental differentiation among the landscapes already existed at the base of the food webs. As animal collagen averages the dietary signals, the distinction among the collagen data themselves is clearer than the distinction among the plants.

### Isotopic differences between animal and human bone collagen

The archaeological record, including the scarcity of settlement remains, has long been interpreted to indicate that, during the Bronze Age, pastoralism was the predominant subsistence system in the North Caucasus [15, 20]. In the following, we assess whether domestic herbivore meat, milk and dairy products were the major protein sources in the human diet. In this case, humans should have been up to one trophic level above the domestic herbivores from the same regions isotopically, i.e. the human bone collagen should have up to 2 ‰ higher δ^13^C values [69] and up to 3 to 6 ‰ (average 4 ‰) higher δ^15^N values than the bones of the domestic animals [77, 78]. The remarkably different Δδ^13^C_human-animal_ and Δδ^15^N_human-animal_ values from these numbers suggest that other food stuffs than meat and secondary animal products contributed significantly to the human diets.

**Fig 9:**
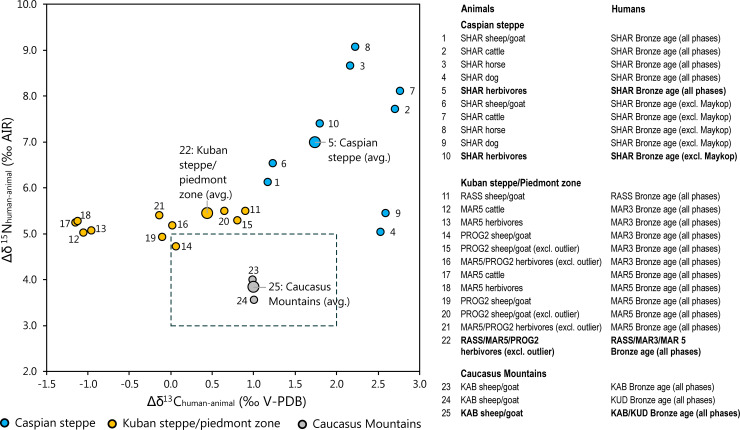
Differences between δ^13^C and δ^15^N values of bone collagen of humans and animals of different species from the Caucasus Mountains, Kuban steppe/piedmont zone, and the Caspi steppe (cf. S2 Table). The box indicates the typical isotopic spacing between two adjacent trophic levels. Δδ^13^C_human-animal_ and Δδ^15^N_human-animal_ values within that range are in agreement with a human diet based on the respective animal sources. The numbers in the graph correspond to the numbers in the list of paired animal and human datasets to the right.

Our considerations are based on individuals older than the age group infans I, because the isotopic composition of their bone collagen may have been biased towards the higher δ^15^N and δ^13^C values of breastmilk [121], even though this distinction is not clearly visible in the present dataset. S2 Table and Fig 9 summarize the Δδ^13^C_human-animal_ and Δδ^15^N_human-animal_ values for different animal species from the same sites or regions. At the upland sites, the human bones had on average 1 ‰ higher δ^13^C values and 3.8 ‰ higher δ^15^N values than the sheep/goat bones from KAB, though this is a rough estimate due to the small sample size. These numbers argue for dietary compositions typical for pastoral communities with substantial contributions of animal-derived food sources, such as meat, milk, and dairy products. The isotopic compositions of the plants from upland locations [17] confirm this interpretation. Assuming that humans would have exploited plants with similar isotope compositions to those reported in Shishlina et al. [17], a primarily vegetarian diet should have resulted in lower δ^15^N values than those recorded for the human collagen from KAB and KUD.

In the Kuban steppe and piedmont area, the humans (RASS, MAR3, MAR5) had on average 0.4 ‰ higher δ^13^C and 5.5 ‰ higher δ^15^N values than the domestic herbivores from these environments (RASS, MAR5, PRO). The high δ^13^C values of the cattle from MAR5 caused some variation among the Δδ^13^C_human-animal_ values. The higher δ^13^C values of the collagen of these animals compared to the humans from the sites in the piedmont zone and Kuban steppe suggest that meat and milk of animals who foraged on a mixture of C_3_ and C_4_ plants did not contribute substantially to the human diet. A looped bronze nose ring and a possible yoke associated with the bucranium of grave 25, which yielded the highest δ^13^C value, indicates that the respective cattle was used as a draught animal [45]. It is a possible example of cattle who may have had access to forage that differed from that available to herds that were generally exploited for human consumption.

Excluding the cattle bones from MAR5, the Δδ^13^C_human-animal_ represented mainly sheep and goat and were within the range to be expected if animal-derived foodstuffs contributed substantially to the human diet. The average Δδ^15^N_human-animal_ values were at the upper end or higher than the typical isotope spacing between members of adjacent trophic levels. This outcome may indicate a human diet nearly exclusively based on animal-derived products. However, the higher average Δδ^15^N_human-animal_ value compared to the mountains suggests a difference in the average compositions of human diets between both regions. The human diet in the piedmont zone and Kuban steppe may have included foodstuffs with higher δ^15^N values than those derived from the exploitation of the herbivore herds. Modern plants growing in the piedmont area and Kuban steppe occasionally contain nitrogen with δ^15^N values of above 7 ‰. These are high enough to have potentially contributed to the elevated δ^15^N values found in the human collagen [17] (Fig 7B and 7F). Such values are, however, the minority among the reported data and occurred in plants such as *Calamagrostis* (reed grass), *Artemisia*, and *Sorbus aucuparia* (rowan). They document a potential general existence of plants with comparatively high δ^15^N values in the landscape in question, albeit in plants which did not necessarily contribute directly to the human diet and with the uncertainties regarding comparing modern and ancient data.

Another possible food source with elevated δ^15^N values is the meat of calves and lambs. Due to isotope fractionation in mammalian milk, meat of unweaned animals tends to have higher δ^13^C and δ^15^N values than that of adult ones [122]. Targeted culling of suckling animals as part of herd management strategies and for food provision of the human groups may have contributed such meat to the human diet, as it is indicated e.g. by the kill-off patterns of LBA animal bone assemblages from mountain settlements [46, p. 14–16, 123]. Indeed, skeletal remains from subadult animals formed a significant portion of the faunal assemblages from the kurgans.

At SHAR in the dry steppe, the humans had on average 1.7 ‰ higher δ^13^C and 7.0 ‰ higher δ^15^N values than the herbivores (S2 Table, Fig 9). The Δδ^15^N_human-animal_ values were larger for all species than what can be expected for representatives of adjacent trophic levels. Also, they varied from the lowest levels for sheep/goat to the highest levels for horses. Δδ^13^C _human-animal_ values also differed among species. For horses and cattle, they again exceeded the expectations for members of adjacent trophic level. Overall, in the dry steppe, the δ^13^C and δ^15^N values of the humans were even more elevated than was to be expected in pastoral communities whose members lived primarily on foodstuffs derived from herbivorous animals. As already discussed for the more humid portions of the steppe, meat of unweaned animals may have contributed significantly to the protein supply of the human diet. In the dry steppe, moreover, plants may also have provided valuable foodstuffs. Shishlina et al. [17] documented the general existence of C_4_ plants with elevated δ^13^C values and reported numerous samples of modern and especially archaeological plants with elevated δ^15^N levels. The comparatively high δ^15^N values of above 10 ‰ occurred particularly in burned grasses and reed mats, which do not represent specific human food plants nor even their edible parts. However, they indicate that–in general–aridity, high temperatures, and partly saline soil conditions in the Kalmykian steppe may have caused δ^15^N values of plants to increase and reach ranges that are elsewhere typical for representatives of higher trophic levels.

Moreover, fish consumption has already been considered in earlier investigations [11, 15, 124]. The steppe is traversed by numerous water courses and freshwater and saline lakes in which fish, especially sturgeons, which provide larger portions of meat, were a widely available food source. Fish are common sources of protein with elevated δ^15^N values, while their δ^13^C values can be highly variable [125, 126]. In the study area, fish consumption is generally likely, whereas the lack of specific analytical data for modern and archaeological fish collagen prevents more precise conclusions about their contribution to the human diet.

### General indications for the human diet

Overall, environmental conditions as well as dietary habits influence the C and N isotope compositions of representatives of all trophic levels. There is a regional trend of increasing δ^13^C and δ^15^N values in both the animal and the human bone collagen from the mountains towards the dry steppe. The tendency is, however, stronger for the humans than for the animals. The isotope composition of the human samples from the uplands is in general agreement with a largely animal-based subsistence and corresponds to expectations for communities with a largely pastoral lifestyle. In the steppe, herbivore meat, milk and dairy products were also crucial for the human diet, and sheep and goat are in better agreement with providing human food sources than products derived from cattle or horses. Nevertheless, the data point to substantial contributions of other sources of protein, and their shares likely grew larger with increasing aridity of the landscape. Such foodstuffs included meat of infant animals, fish, or plants with high δ^13^C and δ^15^N values, as they were detected among the modern vegetation as well as archaeological macro remains.

### Chronological and status-related variation within burial mounds

Sampling of the complete skeletal inventories from several kurgans offered the possibility to explore regional and chronological trends that were previously postulated based on subsamples from different sites [11, 14, 15] as well as connections between dietary composition and the social status of the individuals as inferred from the mortuary context. Moreover, aDNA analyses identified so called “Steppe” and “Caucasus” genetic ancestry among burials from the study area [7]. In the following, we examine possible connections between dietary habits and subsistence strategies as deduced from the light stable isotope compositions of the human collagen and chronological and genetic affiliation as well as social status of the sampled individuals.

### Diachronic change

During the Maykop period, there is archaeological evidence of a multi-component economy that included dry farming and animal husbandry in the piedmont zone and a mobile component that practiced seasonal movements to exploit pastures in the dryer portions of the steppe. The isotope composition of the human collagen of the Maykop period as a whole was highly heterogeneous and covered the complete range of δ^13^C and δ^15^N values observed in this study. There was, however, indication for some differentiation within the Maykop period. The isotope data assigned to the contemporaneous Early Steppe Maykop contexts at SHAR (dry steppe) and Early Maykop contexts at MAR5 (piedmont zone; 3900 and 3400 cal. BCE; Figs 3 and 4) revealed very similar C and N isotope compositions in bone collagen despite the geographic distance between the sites. The pattern is complemented by genetic evidence for “Steppe ancestry” of one individual from each of these sites [7]. The observations from this dataset may be assessed as supporting the archaeological indication of connected communities in the piedmont area and in the dry steppe. Taking published radiocarbon dates and stable isotope data into consideration, however, enlarges the variation of the stable isotope values and indicates that the rather restricted ranges seen in the present study may also be due to the small sample size [11, 14, 127]. Some previously published paired radiocarbon dates of human and terrestrial herbivore bones from the study area revealed remarkable offsets [11, 13, 14, 128]. The extents of these differences ranged from being irrelevant up to several hundred years. A proposed explanation is the consumption of freshwater fish, which introduced carbon with a freshwater reservoir effect to human diets. In the dataset of this study, the difference between the radiocarbon age of a piece of charcoal and a human bone from the Eneolithic burial 37 in kurgan 4 of the site of Progress 2 highlights possible uncertainties regarding the absolute chronological placement of inhumations, which may affect the evaluation of changes of human isotope data over time. We will address freshwater reservoir effects in more detail with additional paired sampling of different materials in future research.

With the transition to the Late Maykop, resp. Late Steppe Maykop cultural periods, the stable isotope data reported here reflect the environmental differentiation between higher δ^13^C and δ^15^N values in the dry steppe and lower δ^13^C and δ^15^N values in the piedmont zone and Kuban steppe. The apparent separation of the drier and more humid portions of the study area regarding dietary signals suggests that mobility ranges of human groups which encompassed both environmental zones or movements of a substantial part of the population from one environmental zone to the other were rare during the Late Maykop period. The indication of dietary differentiation goes along with evidence for genetic heterogeneity, as recently identified using genome-wide analyses [7]. Three individuals of Late Maykop contexts at MAR5 revealed “Caucasus” genetic ancestry, whereas two contemporaneous burials at SHAR exhibited “Steppe ancestry”, resp. an admixture of “Steppe” and “Caucasus” genetic components. Overall, the present data indicate regional differentiation during the Late Maykop period regarding the archaeological record, genetic affiliations, and the origin of the consumed foodstuffs.

The following Middle Bronze Age appeared genetically homogeneous, with burials from SHAR (dry steppe) as well as RASS, MAR3, and MAR5 (Kuban steppe and piedmont zone) being assigned genetic “Steppe ancestry”. The isotope data documented a continued existence of regional differentiation, whereas there were no remarkable alterations over time within the different sections of the steppe. Despite archaeological differentiation into Yamnaya, Novotitorovskaya, Catacomb and North Caucasian groups, the stable isotope composition of the human collagen points to similar dietary habits among the representatives of these entities, that also overlap regarding their radiocarbon dates (Figs 3 and 4). This finding confirms earlier work by [11, 2980, Fig 12], who found evidence for a dichotomy between the northern and southern portions of the steppe and speculated that smaller variation may reflect regional variation among the subsamples from different mounds rather than change over time between about 3500 and 2200 cal. BCE. The datasets of the Middle Bronze Age from single kurgans do neither provide evidence for a gradual change of dietary habits over time from the Yamnaya to the North Caucasian cultures, nor do they clearly support hypotheses regarding shifting mobility strategies between the Yamnaya and Catacomb period [14–17]. The respective models postulated a change from year-round, small scale mobility along the rivers during the Yamnaya period to larger ranges of herd mobility comprising up to 250 km in the Late Catacomb period. This alteration should have been triggered by a climate shift from mild and humid conditions during the Yamnaya period to increasing aridity culminating around 2200 cal. BCE, i.e. during the Late Catacomb. It is supposed to have caused decreasing productivity of the pastures and a necessity of the herds and people to move more often and to cover larger distances [14, 16, 17]. Confirming stable isotope evidence is expected to include overall higher and more variable δ^13^C and δ^15^N values among the representatives of the Catacomb culture compared to those of the Yamnaya culture. The absence of such a trend at SHAR in the Caspian steppe, but also at RASS with much fewer samples, may indicate that the time difference between the two cultures was too short and radiocarbon dating possibly too imprecise due to freshwater reservoir effects [14, 128] to reflect the supposed climate change. Existing trends might also have been superimposed by other factors that influenced the isotope data, such as variation among the isotope composition of the foraged plants [17] and overlap of the data range of different food sources. Moreover, the analysis of soil samples from below several kurgans indicates that the aridisation culminated in the Catacomb and post-Catacomb horizon at around 2200 cal. BCE [57]. Because our dataset is rather limited after ca. 2400 cal. BCE, we may have missed out on some robust evidence for the supposed climate change.

Representatives of the final Middle and the Late Bronze Age come from the upland sites of Kabardinskiy 9 and Kudakhurt and indicate very similar dietary compositions that agree with a pastoral subsistence strategy. The distinction between a genetic “steppe” ancestry of two individuals from Kabardinskiy 9 and “Caucasus” ancestry of two individuals from Kudakhurt (who were not part of the small subsample of this study) is not reflected in the isotope data that relate to dietary compositions. A significant change in food production occurred in the Early Iron Age, when elevated δ^13^C values point to the introduction of C_4_ dietary plants, such as millet. The post-Bronze Age data from this study confirm earlier data from Klin Yar that attested to the establishment of millet cultivation and its contribution to the diet of Iron Age populations [13, 31, 101, 120].

### Social differentiation

Even though burial in a mound is by itself interpreted as indicating an elevated social status of the respective individuals, some of the burials stand out due to the presence of wagons or their generally rich furnishing (Fig 5). We explored the stable isotope data of the bone collagen of these conspicuous individuals for a different, supposedly higher quality average diet, as it was e.g. documented in central European contexts [129]. The analyses did disclose some similarities among the light stable isotope compositions of the bone collagen of these individuals at the same sites (Fig 3). However, they did not show a consistent pattern of particularly high or low levels of either C or N isotope values. Because larger labor input is required to produce foodstuffs from organisms of a higher trophic level, animal-derived food is often considered to be of a higher value than plant-based foodstuffs in agropastoral communities [129]. In contrast, in pastoral societies with generally larger shares of meat and milk/dairy products in the human diet, it may not be the contribution of animal-derived foodstuffs in general, but the kind of meat or dairy products that determined dietary quality. The general assessment of dietary compositions based on the trophic level spacing suggests that δ^15^N values of the human collagen are typically higher than expected for a diet that was primarily based on herbivore meat and dairy products. Therefore, a simplistic assumption according to which higher δ^15^N values imply larger portions of animal-derived foodstuffs and reflect an elevated social status do not apply in the present contexts. Instead, the isotope composition of the human collagen seems to reflect a complex amalgamation of isotope variation among the plants at the base of the food webs and higher trophic level animals as well as the use of multiple and diverse dietary sources such as fish, the meat of unweaned animals or non-domesticated wildlife.

Moreover, besides the social status of an individual during life, multiple other factors may have determined grave furnishing and grave constructions. Material property might have been passed on to the next generation so that a limited number or restricted wealth of grave goods may not necessarily reflect lower social status of the respective individual in lifetime. So far, our study focused on mounds that were often the largest within their respective cemeteries. Future research will include samples from the smaller monuments and explore possible relations between social and dietary variation among burials from the same sites.

## Conclusions

Carbon and nitrogen stable isotope compositions of human and animal collagen from Bronze Age burial contexts in the north Caucasus reflect both environmental conditions and dietary habits of pastoral communities. The analytical data of plants, animals, and humans vary gradually from south to north, from the mountain plateaus, across the piedmont area and humid Kuban steppe, into the dry, almost desert-like portions of the Caspian steppe. This regional differentiation suggests that during all time periods the home ranges of the human communities and their herds remained generally limited to movements within the respective landscapes. Large-scale mobility spanning several hundred kilometers and across multiple environmental zones should have resulted in a less clear isotopic distinction between communities in the mountains and those of the southern and northern parts of the steppes. Only few animal or human individuals revealed stable isotope data that point to a possible origin in a landscape other than that where their skeletal remains were found. The animals do not represent the total range of the isotope data of ancient or modern the vegetation in each landscape, which attests to averaging different environmental signals and selective foraging that restricted the range of isotope signals that entered the Bronze Age food webs.

In addition to the general differentiation of the isotope composition of bone collagen between landscapes, isotope spacing between herbivore and human collagen varied among the three main landscapes or ecozones, which implies different dietary compositions. The isotope signals of humans from the upland locations were about one trophic level above those of the domestic animals, which is largely consistent with pastoral subsistence strategies. In contrast, isotope spacing between herbivores and humans in the humid and–even more so–in the arid areas of the steppe exceeded one trophic level. Among the herbivore species, sheep and goat were more likely the major contributors to the human diet than cattle or horses. Nevertheless, the investigated pastoral communities must have used a variety of food sources in addition to the meat, milk and dairy products produced from their livestock. The isotope composition of modern and archaeological vegetation [17] attested to the general availability of foodstuffs with comparatively high C and N isotope values. Moreover, the meat of juvenile unweaned animals or fish may have contributed significantly, and the portion of these non-herbivorous components was supposedly larger in the dry steppe than in the humid steppe and on the upland plateaus.

Sampling all inhumations with sufficient bone preservation from several mounds revealed only subtle alterations in the stable isotope composition of human collagen over time at the same locations. The data indicate some changes within the Early Bronze Age Maykop period and were largely consistent across the later cultural entities including the Yamnaya, Novotitorovskaya, Catacomb and North Caucasian archaeological cultures. Whether climate change, including increasing aridity peaking around 2200 BCE had a significant impact on the stable isotope composition of the human bone collagen remains disputable. No such indication is noted in the humid areas until ca. 2400 BCE, the region and time that is best represented among our data. This implies largely sustainable subsistence strategies that tolerated unfavorable conditions to a certain extent. Moreover, the observation implies that distinctions in material culture did not necessarily go along with different dietary habits and subsistence strategies.

Despite the variability of the datasets at each of the sites, age, sex and outstanding grave furnishing were not reflected in any systematic grouping of the data. If dietary differences existed among these groups, they were not reflected in the isotope composition of the bone collagen.

The stable isotope data of the present study offered valuable insights into subsistence and lifestyle of the Bronze Age communities in the Northern Caucasus. Future research will build on these data and enlarge the sample size for each time slice of the Bronze Age to reveal a potentially more nuanced picture of dietary alterations over time and add more graduated spatial information from strontium and oxygen isotope compositions of dental enamel. The Bronze Age is widely considered a time of increased mobility that allowed for an inter-regional spread of innovations, raw materials and goods in the North Caucasus area. Archaeogenetic investigations of human burials from central Europe point to substantial population shifts being associated with people who originated in contexts of the Yamnaya culture [90, 91]. On the other hand, the light stable isotope data presented here indicate human home ranges that did not regularly cross the transitions between different environmental zones. Strontium and oxygen isotope data of future studies that are related to geological and climatic conditions will provide essential information to further evaluate questions on human mobility in landscapes that were possible homelands of human groups who contributed substantially to the population genetic history of central Europe.

## Supporting information

S1 Fig Chronological overview of archaeological cultures of the Bronze Age in the North Caucasus as well as easter anatolia and northern mesopotamia. The chrono-cultural synopsis is based on an initial draft by Trifonov 2004 [100] with later modifications [7, 8, 130].
(PDF)

S2 Fig Map of the study area and investigated sites. The pie charts indicate the archaeological cultural affiliations of the sampled individuals at each of the studied sites. The sizes of the pie charts correspond the numbers of sampled individuals. Background map after {Stone, 2003 #9972}. This dataset is openly shared, without restriction, in accordance with the NASA Data and Information Policy.
(EPS)

S3 Fig Eneolithic burial Progress 2, Grave 37 *in situ*. The body of the male individual was covered with red ochre. Grave goods comprised a long flint blade, a flint adze, a flint projectile head and another flint object.
(TIF)

S4 Fig Sharakhalsun 6, Kurgan 2, Grave 18. The dead body was placed in a sitting position onto a wooden four-wheeled wagon in a deep catacomb-like burial chamber with entrance pit. The skeleton exhibited traces of numerous pre- and perimortem injuries.
(TIF)

S5 Fig Rasshevatskiy 1, Kurgan 21, Grave 7. Burial of five individuals in a pit with a rectangular shaft with a step on which a 4-wheeled dismantled wagon was deposed.
(TIF)

S6 Fig Marinskaya 5, Grave 25. Disturbed inhumation burial in a square chamber surrounded by wooden posts and covered by wooden planks. The chamber and related mound embankment was covered by a stone packing. The inventory included 2 bronze daggers, 1 golden ear-ring, 1 ceramic vessel, 1 bone arrowhead. Outside the chamber, there was a deposit of two bucrania with nose rings and a badly preserved wooden object (yoke?, fixing pole?).
(TIF)

S1 Table A. Archaeological contexts as well as carbon and nitrogen stable isotope data of human remains. The table lists short descriptions of the grave features, burial inventory, archaeological cultural affiliation, radiocarbon and dendrochronological dates, sampled skeletal element, results of the elemental and stable isotope analysis, age and sex information as well as ancestry groups, mtDNA and Y-chromosome haplogroups according to Wang et al. [7] of each burial. B. Archaeological contexts as well as carbon and nitrogen stable isotope data of animal remains. The table lists the contextual information, animal species, archaeological cultural information, radiocarbon dates, skeletal element as well as the results of the elemental and stable isotope analysis of each analyzed specimen.
(XLSX)

S2 Table Average δ^13^C and δ^15^N values of animal and human bone collagen and human-animal-differences of the average isotope compositions. The animal data are grouped according to environmental zones, sites and species.
(XLSX)
