# Supplementary figures and images for: Diet and subsistence in Bronze Age pastoral communities from the southern Russian steppes and the North Caucasus
Source: PLoS One. 2020 Oct 14;15(10):e0239861. doi: 10.1371/journal.pone.0239861 (PMC7556513; doi:10.1371/journal.pone.0239861)

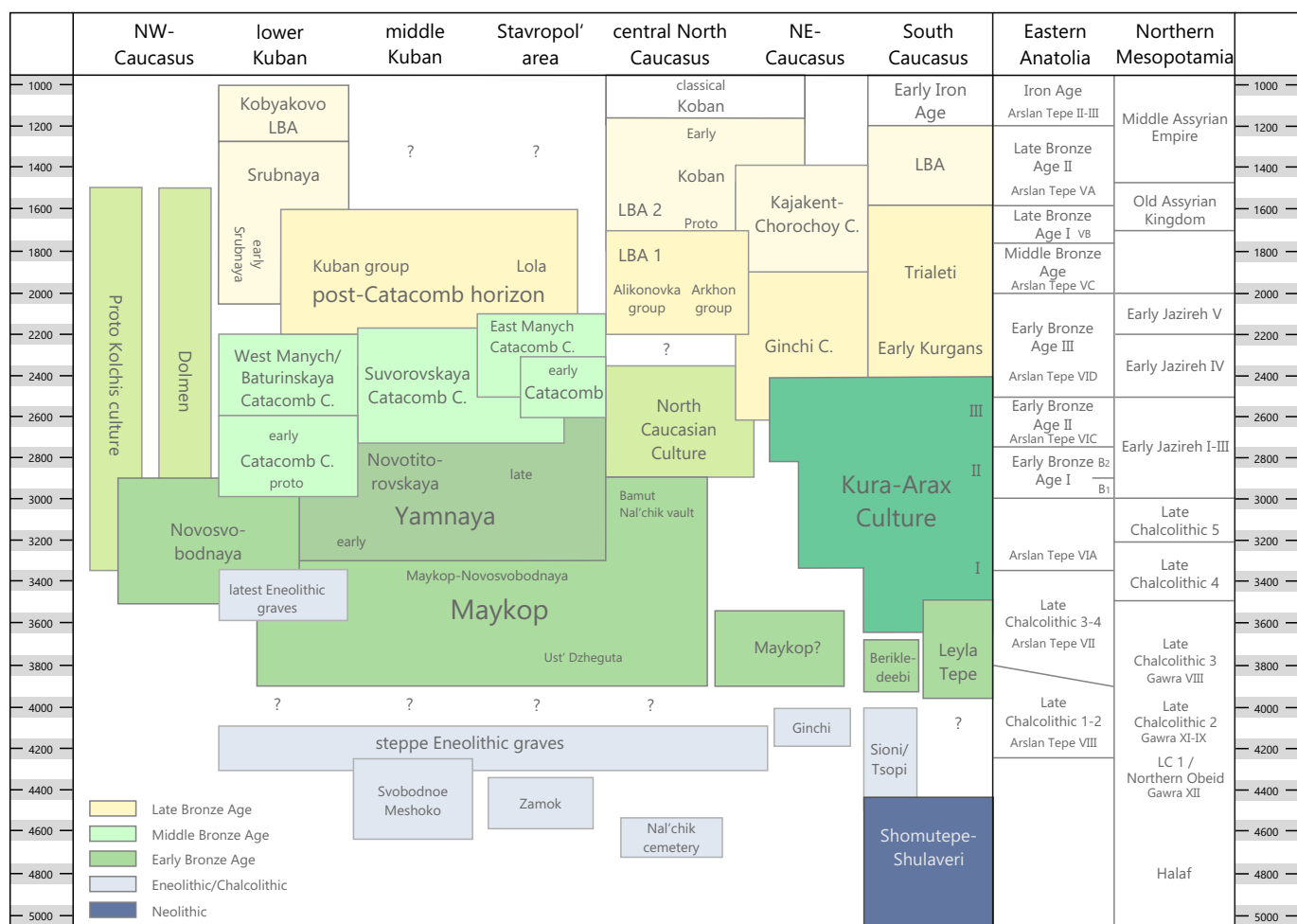

Supplement: S1 Fig — The chrono-cultural synopsis is based on an initial draft by Trifonov 2004 [100] with later modifications [7, 8, 130]. (PDF) [file pone.0239861.s001.pdf]
